# Magnetic-field-driven targeting of exosomes modulates immune and metabolic changes in dystrophic muscle

**DOI:** 10.1038/s41565-024-01725-y

**Published:** 2024-07-22

**Authors:** Chiara Villa, Valeria Secchi, Mirco Macchi, Luana Tripodi, Elena Trombetta, Desiree Zambroni, Francesco Padelli, Michele Mauri, Monica Molinaro, Rebecca Oddone, Andrea Farini, Antonella De Palma, Laura Varela Pinzon, Federica Santarelli, Roberto Simonutti, PierLuigi Mauri, Laura Porretti, Marcello Campione, Domenico Aquino, Angelo Monguzzi, Yvan Torrente

**Affiliations:** 1 Stem Cell Laboratory, Dino Ferrari Center, Department of Pathophysiology and Transplantation, Università degli Studi di Milano, Milan, Italy; 2 Department of Materials Science, University of Milano Bicocca, Milan, Italy; 3 NANOMIB, Nanomedicine Center, University of Milano Bicocca, Milan, Italy; 4 Luxembourg Centre for Systems Biomedicine, Department of Biomedical Data Science, Luxembourg City, Luxembourg; 5 Flow Cytometry Service, Clinical Pathology, Fondazione IRCCS Ca’ Granda Ospedale Maggiore Policlinico, Milan, Italy; 6 Advanced Light and Electron Microscopy Bioimaging Center ALEMBIC, San Raffaele Scientific Institute – OSR, Milan, Italy; 7 Department of Neuroradiology, IRCCS Foundation Neurological Institute ‘Carlo Besta’, Milan, Italy; 8 Neurology Unit, Fondazione IRCCS Ca’ Granda Ospedale Maggiore Policlinico, Milan, Italy; 9 National Research Council of Italy, Proteomics and Metabolomics Unit, Institute for Biomedical Technologies, ITB-CNR, Segrate, Milan, Italy; 10 Clinical Proteomics Laboratory, ITB-CNR, CNR.Biomics Infrastructure, Elixir, Milan, Italy; 11 Veterinary Medicine, Department Clinical Sciences, Equine Sciences, Equine Musculoskeletal Biology. Utrecht University, Utrecht, Netherlands; 12 Department of Earth and Environmental Sciences, University of Milano Bicocca, Milano, Italy

**Keywords:** Nanoparticles, Tissue engineering and regenerative medicine, Nanobiotechnology

## Abstract

Exosomes are promising therapeutics for tissue repair and regeneration to induce and guide appropriate immune responses in dystrophic pathologies. However, manipulating exosomes to control their biodistribution and targeting them in vivo to achieve adequate therapeutic benefits still poses a major challenge. Here we overcome this limitation by developing an externally controlled delivery system for primed annexin A1 myo-exosomes (Exo^myo^). Effective nanocarriers are realized by immobilizing the Exo^myo^ onto ferromagnetic nanotubes to achieve controlled delivery and localization of Exo^myo^ to skeletal muscles by systemic injection using an external magnetic field. Quantitative muscle-level analyses revealed that macrophages dominate the uptake of Exo^myo^ from these ferromagnetic nanotubes in vivo to synergistically promote beneficial muscle responses in a murine animal model of Duchenne muscular dystrophy. Our findings provide insights into the development of exosome-based therapies for muscle diseases and, in general, highlight the formulation of effective functional nanocarriers aimed at optimizing exosome biodistribution.

## Abstract (web summary)

Exosome targeting for therapeutic needs remains a challenge. Here, the authors show that ferromagnetic-nanotube-passivated exosomes promote the transition of proinflammatory macrophages to an anti-inflammatory state and myogenic maturation of dystrophic muscle progenitors in a murine model.

## Main

Timely resolution of inflammation is necessary to restore muscle homeostasis following infection or damage and avoid chronic inflammatory pathologies, including muscular dystrophies. Macrophages are key players in this process due to their capacity to transition from a generally proinflammatory state to an anti-inflammatory phenotype. Engineered exosomes, extracellular vesicles <200 nm in size^1,2^, can carry multiple signalling biomolecules, including pro-resolving immune mediators, such as annexin A1 (ANXA1), which has emerged as a key regulator of macrophage polarization^2,3^. Previous studies of exosome delivery for treatment of different forms of muscular dystrophy have had promising results^4–6^, suggesting that accurate manipulation of exosomes can facilitate tissue repair during pathological processes. However, systemic biodistribution is challenging due to the influence of exosomal composition, in particular the lipid and protein content, on pharmacokinetics and bioavailability^2,7–9^.

A potential solution to this issue is the use of exosome carriers that can be controlled by external stimuli for accurate targeting and delivery, allowing on-demand manipulation of the biodistribution of therapeutic exosomes^10^. We propose using ferromagnetic nanotubes (NT-MAGs) for magnetic-field-controlled exosome delivery and localization in vivo. The NT-MAGs are primed with surface-anchored ANXA1 myo-exosomes (Exo^myo^) by exploiting a Coulombic interaction. We demonstrate that the NT-MAGs can effectively bind and transport Exo^myo^ and, after systemic injection, accumulate in designated muscles in a murine model of Duchenne muscular dystrophy (DMD), the mdx mouse^11^, upon application of an external magnetic field. This controlled delivery maximizes the biological functions of Exo^myo^ in dystrophic muscles, leading to improved muscle function.

## Results

### Improved muscle function with serial exosome injection

**Fig. 1:**
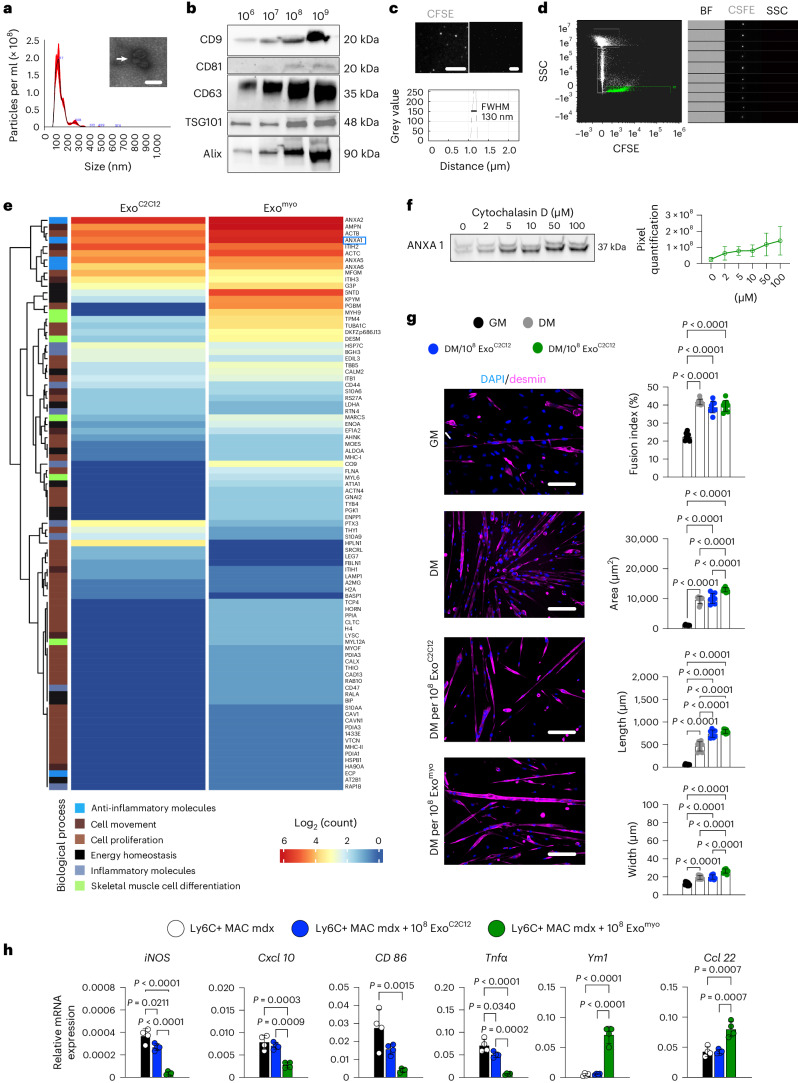
Exo^myo^ enhances myogenesis and switches macrophages to an anti-inflammatory state. **a**, NTA and TEM (inset; the arrow indicates exosomes) were performed on Exo^C2C12^ obtained through differential ultracentrifugation (average diameter, 137.5 nm; mode, 116 nm; s.d., 56.2 nm). Scale bar, 200 nm. **b**, Western blot of CD9, CD81, CD63, TSG101 and Alix in 10^6^, 10^7^, 10^8^ and 10^9^ Exo^C2C12^ samples. **c**, Representative super-resolution dSTORM reconstructed image showing CFSE-labelled exosomes from C2C12 (upper left panel) and the FWHM of the fluorescence intensity distribution (lower panel) obtained from dSTORM imaging (upper right panel). Scale bars, 10 μm (left) and 1 μm (right). **d**, Representative Amnis ImageStream of CFSE-labelled Exo^C2C12^ showing the scatter plot of SSC versus CFSE intensity, which resolves three discrete populations (SpeedBeads (SB); Background (BG); Exosomes (R1), 0–200 nm) (left panel). CFSE staining is evident only in the R1 Exo^C2C12^ population as confirmed by CFSE, SSC and bright-field (BF) imagery (right panel). In **a**–**d** representative images from *n* = 3 independent experiments are shown. **e**, Heatmap of the top 100 differentially expressed proteins between the Exo^C2C12^ and Exo^myo^ groups. *n* = 3 samples per group. **f**, Western blotting (left panels) and quantification (right panels) of ANXA1 expression in exosomes isolated from cytochalasin D-primed C2C12 cells. Cells were primed with escalating cytochalasin D doses (0, 2, 5, 10, 50, 100 μM). Blots are representative of *n* = 3 independent experiments; data are presented as mean ± s.d. of *n* = 3 independent experiments. **g**, Myogenic commitment of desmin-positive mdx SCs cultured for 24 h in growth medium and for 7 days in differentiation medium (DM), DM supplemented with 10^8^ Exo^C2C12^ or DM supplemented with 10^8^ Exo^Myo^. Scale bars, 75 μm. Graphs show quantification of the fusion index and myotube area, length and width. Data are presented as mean ± s.d.; *n* = 2 or 3 independent experiments, for each experiment *n* = 3 or 4 images per group were quantified; ordinary one-way ANOVA followed by post hoc Tukey multiple-comparison test. **h**, Quantitative reverse-transcription PCR transcript levels of the indicated genes were quantified from freshly purified (by FACS) circulating macrophages (iMACs) and iMACs cultured for 24 h in the presence of 10^8^ Exo^C2C12^ or 10^8^ Exo^myo^. Data are presented as mean ± s.d.; *n* = 4 independent experiments; ordinary one-way ANOVA followed by post hoc Tukey multiple-comparison test. Source data

Exosomes derived from myogenic cells can elicit functional improvements and alleviate muscle wasting in animal models of muscular dystrophy, sarcopenia and cachexia^12,13^. In contrast, endogenously secreted exosomes may have a protective role or contribute to spreading pathology in DMD^14^. We collected exosomes from C2C12 cell culture medium (Exo^C2C12^) following the MISEV2018 guidelines^15^. Nanoparticle tracking analysis (NTA) and transmission electron microscopy (TEM) of Exo^C2C12^ revealed the presence of cup-shaped vesicles, characteristic of exosomes^15^ (Fig. 1a). Western blotting confirmed the expression of markers related to exosomal tetraspanins and proteins crucial for exosome formation (Fig. 1b). The absence of non-exosomal markers excluded potential contaminants or non-exosomal elements (Supplementary Fig. 1a). CFSE staining of Exo^C2C12^ was confirmed by stochastic optical reconstruction microscopy (dSTORM) (Fig. 1c) and Amnis imaging flow cytometry (Fig. 1d).

Liquid chromatography–tandem mass spectrometry (LC–MS/MS) of Exo^C2C12^ revealed the presence of proteins belonging to Gene Ontology (GO) pathways involved in cell proliferation and movement, skeletal muscle cell differentiation, energy homeostasis, and inflammatory and anti-inflammatory processes (Fig. 1e). Among the most expressed proteins, ANXA1 plays a major role in promoting resolution of the inflammatory state, neutrophil apoptosis and rewiring macrophages towards an anti-inflammatory phenotype with enhanced efferocytosis capacity^16–20^. Using cytochalasin D as a cytoskeletal perturbator^18,21^, C2C12 cells were primed in vitro. ANXA1 expression linearly increased across increasing concentrations of cytochalasin D (Fig. 1f). Proteomic analysis confirmed the increased expression of exosomal ANXA1 when using 100 μM cytochalasin D (Fig. 1e).

To investigate the role of Exo^myo^ in myogenic fate decisions in dystrophic muscle cells, satellite cells (SCs) were isolated from the hind limbs of 3-month-old mdx mice and induced to differentiate into myotubes (Fig. 1g). Exo^myo^ fostered the development of myotubes with a greater length and width than Exo^C2C12^ or medium alone. This suggests the participation of Exo^myo^ in later stages of myogenic differentiation (Fig. 1g). In parallel, freshly isolated macrophages from the muscles of 3-month-old mdx mice were cultured for 5 days in the presence of 10^8^ Exo^myo^ or 10^8^ Exo^C2C12^. Untreated macrophages expressed elevated levels of canonical inflammatory M1 genes^22^. In contrast, macrophages treated with Exo^myo^ exhibited heightened expression of anti-inflammatory M2-associated genes^22^ and concurrent downregulation of M1 genes compared with macrophages treated with 10^8^ Exo^C2C12^ (Fig. 1h and Supplementary Discussion 1). These findings provide compelling evidence of the induction of M2 macrophage polarization by Exo^myo^.

**Fig. 2:**
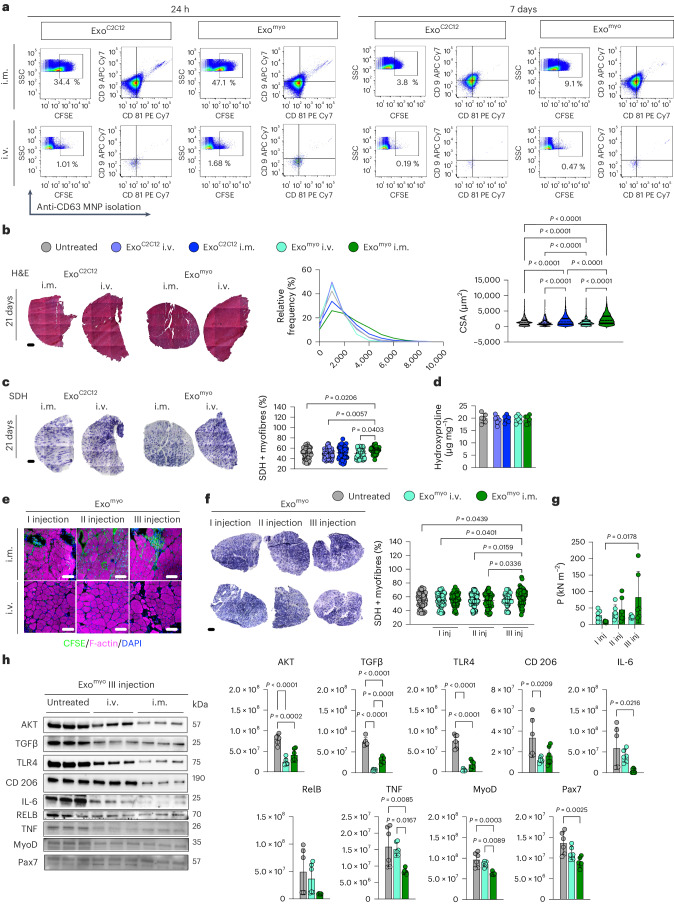
Repeated intramuscular injection of Exo^myo^ improves muscle function in mdx mice. Mdx mice were injected intramuscularly (i.m.) or intravenously (i.v.) with 10^9^ Exo^C2C12^ or 10^9^ Exo^myo^. The TA was assessed 24 h, 7 days and 21 days after treatment. For each administration route, *n* = 6 mice per Exo^C2C12^ or Exo^myo^ were used for each time point; *n* = 6 untreated mice were used as controls. **a**, Exosomes were immunomagnetically isolated from dissociated muscle tissues using anti-CD63 MNP. Isolated CD63+ exosomes were characterized by FACS for the expression of exosomal markers. Left, Representative FACS dot-plots show the proportion of muscle-isolated CFSE+ exosomes in both Exo^C2C12^- and Exo^myo^-injected mice. Right, Representative FACS dot-plots show the coexpression of CD9 and CD81 within the CFSE+ Exo^C2C12^ and Exo^myo^ populations. FACS was performed on *n* = 3 mice per group (pooled muscles) in *n* = 2 independent experiments. **b**, Representative H&E and SDH staining images from the TA muscles of mdx mice 21 days after intramuscular and intravenous injection of Exo^C2C12^ or Exo^myo^. Scale bars, 200 μm. **c**, Quantification of the myofibre area and relative frequency distribution of the myofibre CSA in the TA 21 days after intramuscular and intravenous administration of Exo^C2C12^ or Exo^myo^. For each group, H&E images were counted from *n* = 5 mice per group: *n* = 5,677 myofibres for untreated mdx (median, 1,301.56 μm^2^; 25th percentile, 702.46 μm^2^; 75th percentile, 2,074.69 μm^2^); *n* = 5,562 for intramuscular Exo^C2C12^ (median, 1,535.042 μm^2^; 25th percentile, 781.50 μm^2^; 75th percentile, 2,574.76 μm^2^) and *n* = 4,581 for intravenous Exo^C2C12^ (median, 1,156.076 μm^2^; 25th percentile, 719.19 μm^2^; 75th percentile, 2,074.90 μm^2^); *n* = 5,455 for intramuscular Exo^myo^ (median, 2,057.711 μm^2^; 25th percentile, 1,052.32 μm^2^; 75th percentile, 3,356.76 μm^2^) and *n* = 5,326 for intravenous Exo^myo^ (median, 1,116.286 μm^2^; 25th percentile, 658.91 μm^2^; 75th percentile, 1,765.78 μm^2^). For morphometric analysis, images were quantified using ImageJ software. Violin plots showing CSA median (dotted lines) and quartiles (solid lines); Kruskal–Wallis with Dunn’s multiple-comparison test. **c**, Representative SDH staining and quantification of the percentage of SDH+ myofibres in the TA 21 days after intramuscular and intravenous administration of Exo^C2C12^ or Exo^Myo^. *n* = 4 mice per group, *n* = 16 slices per mouse. Scatter dot-plots showing SDH+ fibre percentage as mean ± s.d.; Kruskal–Wallis with Dunn’s multiple-comparison test. **d**, Fibrosis was quantified in TA by the hydroxyproline assay 21 days after intramuscular and intravenous administration of Exo^C2C12^ or Exo^myo^. Data are presented as mean ± s.d. of *n* = 3 samples per group in two independent experiments; one-way ANOVA followed by post hoc Tukey multiple-comparison test. **e**, Three-month-old mdx mice were treated with one (I), two (II) or three (III) 10^9^ Exo^myo^ intramuscular (*n* = 12 mice) and intravenous (*n* = 6 mice) injections, once a week. A control untreated group was used as control (*n* = 6 mice). Mice were killed 30 days after the first injection. Representative images of F-actin+ muscle cells (red) with Exo^myo^ (green). Scale bars, 50 μm. **f**, Representative images of SDH staining and quantification of SDH+ myofibres. Scale bars, 200 μm. Scatter dot-plots showing mean ± s.d. of *n* = 4 mice per group, *n* = 16 slice per mouse; Kruskal–Wallis with Dunn’s multiple-comparison test. **g**, Tetanic force of TA muscles from mdx mice injected intramuscularly and intravenously with the three different Exo^myo^ doses, 30 days after the first injection. Data are presented as mean ± s.d. of *n* = 6 mice per group; two-way ANOVA followed by post hoc Tukey multiple-comparison test. **h**, Cropped representative images of Western blots showing the expression of proteins involved in inflammation and fibrosis in TA muscles from mdx mice treated with three (III) injections of Exo^myo^. Data are presented as mean ± s.d. *n* = 3 mice per group in two independent experiments; one-way ANOVA followed by post hoc Tukey multiple-comparison test. Source data

To investigate the muscle-targeting ability of Exo^myo^ in dystrophic mice, we labelled 10^9^ Exo^myo^ and 10^9^ Exo^C2C12^ with carboxyfluorescein succinimidyl ester (CFSE) and injected them into the tibialis anterior (TA) muscle of mdx mice intramuscularly or intravenously. The intramuscular injection resulted in a higher percentage of CFSE+ exosomes than intravenous delivery (Fig. 2a and Supplementary Discussion 2), confirming the rapid clearance and low accumulation of systemically injected exosomes in target muscles^23^.

Histological analysis revealed a significant increase in myofibre cross-sectional area (CSA) with intramuscular injection of Exo^myo^ versus Exo^C2C12^, intravenous administration of Exo^myo^ or Exo^C2C12^, or untreated mdx mice (Fig. 2b). In addition, intramuscular Exo^myo^ increased the percentage of oxidative succinate dehydrogenase (SDH)+ myofibres in mdx mice compared with intramuscular Exo^C2C12^ and intravenous administration (Fig. 2c). Fibrosis was not different between the tested groups (Fig. 2d). Thus, Exo^myo^ triggers metabolic reprogramming in dystrophic muscles after direct injection.

To boost the functional delivery of Exo^myo^, mdx mice were given up to three doses of exosomes, once a week. The bioavailability of CFSE-labelled Exo^myo^ was determined by ex vivo optical bioluminescence imaging 21 days after the first injection. CFSE-Exo^myo^ was detectable in the TA only after direct injection; systemic injections led to accumulation of CFSE-Exo^myo^ in filter organs (for example, liver and spleen; Supplementary Fig. 1b). Immunofluorescent staining of dystrophic muscle injected with Exo^myo^ confirmed the longer retention of CFSE with increasing number of doses. Accumulation was observed around F-actin-positive muscle fibres and inside bona fide regenerative small fibres (Fig. 2e). Analysis of SDH immunoreactivity in muscle revealed a significant increase in oxidative SDH+ fibres after the third intramuscular Exo^myo^ injection compared with intravenous injection at all time points (Fig. 2f). A significant increase in tetanic force was observed in dystrophic muscle treated with three serial intramuscular injections of Exo^myo^ versus intravenous injection (Fig. 2g).

To determine whether the enhanced muscle force and metabolic remodelling were determined by reduced inflammation, we surveyed the protein levels of proinflammatory M1 (tumour necrosis factor (TNF), interleukin 6 (IL-6)), anti-inflammatory M2 (transforming growth factor-β (TGFβ), CD206) and innate immune memory (TLR4/AKT/RELB) markers of macrophages in TA muscle from Exo^myo^-treated mdx mice (Fig. 2h). Three doses of Exo^myo^ resulted in a notable reduction in TNF and IL-6 (Fig. 2h) concomitant with diminished activity in the TLR4/AKT/RELB cascade and decreased expression of CD206. There was a concurrent reduction in TGFβ, contributing to pathologic muscle fibrosis (Fig. 2h). Collectively, these findings indicate attenuation of the dystrophic inflammatory milieu following repeated intramuscular administration of Exo^myo^. In addition, three doses of Exo^myo^ may reduce the extrinsic signalling activating SCs, as indicated by the decrease in MYOD and PAX7 protein expression (Fig. 2h). In muscle injected with three doses, 25% of CD45+ cells coexpressed macrophage markers CD68 and CFSE, whereas 2.9% of CD45− cells expressed the SC markers integrin-α7 and CFSE (Supplementary Fig. 1c). These findings support the hypothesis that preferential uptake of Exo^myo^ from immune system cells and tissue-confined Exo^myo^ enrichment may be responsible for driving macrophage immunomodulation, ameliorating skeletal muscle metabolism and improving muscle force in dystrophic mdx mice.

### Fabrication of exosome nanocarriers

**Fig. 3:**
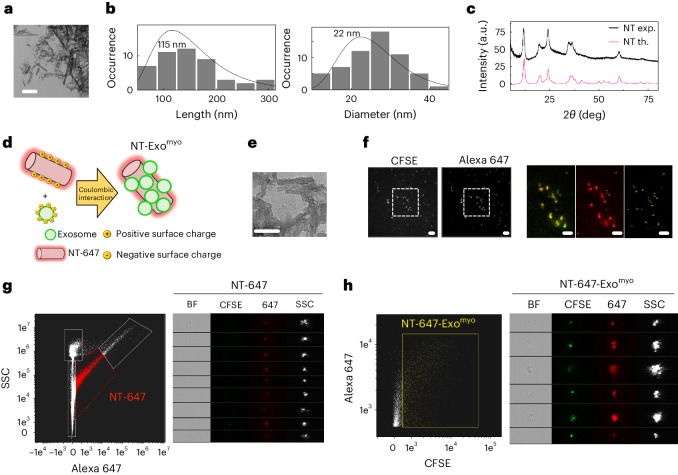
Fabrication of exosome nanocarriers. **a**, TEM of bare NTs. Scale bar, 200 nm. **b**, Size distribution of NT length (left) and diameter (right) obtained from the analysis of TEM images. The solid line is the fit of the size distribution with a log-normal distribution of the indicated average value. **c**, Powder X-ray diffraction patterns of bare NTs (exp.) and simulated crystalline chrysotile NTs (th.). **d**, Sketch of the ionic self-assembly mechanism exploited to attach the conjugated dye Alexa Fluor 647 to the surface of NTs decorated with CFSE + Exo^myo^. **e**, TEM of NTs decorated with exosomes. Scale bar, 200 nm. **f**, Representative 100× magnification of NT-647 labelled with Exo^myo^. Scale bars, 1 μm. Dotted areas were analysed by dSTORM (left image for NT-647-Exo^myo^; middle image for Exo^myo^, right image for the dSTORM reconstruction). Scale bar, 1 μm. **g**, Amnis ImageStream of NT-647 showing the scatter plot of SSC versus Alexa 647 intensity, which resolves four discrete populations (SpeedBeads (SB); Background (BG); NT-647; NT-647 aggregates) (left panel) and representative Amnis flow cytometry image gallery of NT-647 (red) (right panel). **h**, Amnis ImageStream of dual-functionalized NT-647-Exo^myo^ (yellow) showing the scatter plot of CFSE versus Alexa 647 intensity (left panel) and representative Amnis imaging flow cytometry image gallery of NT-647-Exo^myo^.

Synthetic hydrated magnesium silicate (Mg_3_(Si_2_O_5_)(OH)_4_) nanotubes (NTs) were fabricated under hydrothermal conditions, followed by a multistep chemical treatment to generate alternating layers of silica and brucite in a tubular configuration (Fig. 3a). TEM and X-ray diffraction confirmed that crystalline NTs with a length of 115 nm and diameter of 22 nm were obtained (Fig. 3b,c). The brucitic external surface induces a positive *Z*-potential, allowing the binding of anionic species through an ionic self-assembly (ISA) scheme^24^. The exosome surface is negatively charged by the presence of glycosylated proteins^25^, enabling the exosome to anchor to the NT surface in an aqueous environment (Fig. 3d). Exosomes were effectively immobilized onto the NT surface (Fig. 3e). The colocalization and stability of Alexa Fluor 647-stained NTs (Supplementary Fig. 2a–c) carrying Exo^myo^ were demonstrated directly in buffer solution by dSTORM (Fig. 3f)^26^ and Amnis imaging flow cytometry (Fig. 3g,h)^27^.

**Extended Data Fig. 1:**
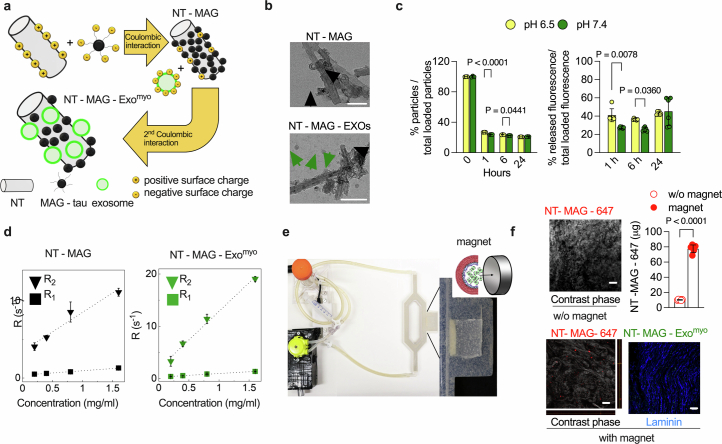
Magnetic targeting ability of exosome nanocarriers in vitro. **a**, Schematic of the two-step ionic self-assembly mechanism exploited to attach ferromagnetic nanoparticles (MAG-tau) and ExoMyo to the surface of nanotubes (NTs) to obtain multifunctional nanocarriers (NT-MAG-Exomyo). **b**, Transmission electronic microscopy (TEM) images of NTs functionalized with MAG-tau (top, NT-MAG, scale bar = 50 nm) and of NT-MAG-Exomyo (bottom, scale bar = 100 nm). Black and green arrows indicate the MAG-tau nanoparticles and exosomes, respectively. **c**, Exomyo release dynamic as a function of the environmental pH in buffer solution. Data are shown as mean ± SD; n = 3 independent experiments; two-way ANOVA followed by the post hoc Tukey multiple comparison test. **d**, Measurement of the magnetic relaxation rates, R2 and R1, as a function of the concentration of NT-MAG (left) and NT-MAG-Exomyo (right) in aqueous suspension. **e**, Digital image of the artificial circulatory system model exploited to investigate the effect of an external magnetic field on the diffusion of NT-MAG-647 and NT-MAG-Exomyo in the blood flux and across a biological tissue barrier. Schematic figure illustrating the arterial wall of C57Bl mice leaned against the reservoir. For magnetic delivery, a 0.5 Tesla ring magnet was applied to the reservoir. **f**, Confocal microscope images showing the localization of NT- MAG- 647 within the arterial elastic lamina (grey for contrast phase image) with and without magnet placement; confocal microscope image of NT-MAG-Exomyo (green) accumulating within the arterial elastic lamina (grey for contrast phase image, blue pseudocolor for laminin staining) 30 min after magnetic targeting. The amount of collected NT-MAG in the reservoir was calculated from the percentage of relative fluorescence. Scale bars = 20 μm. Data are presented as mean ± SD; n = 3 samples per group in two independent experiments; two-tailed unpaired t-test. Source data

The NTs were sensitized to the magnetic field by partially covering their surface with ferromagnetic nanoparticles capped by tetramethyl ammonium 11-aminoundecanoate (TAU; Extended Data Fig. 1a and Supplementary Fig. 2d–f). TEM (Extended Data Fig. 1b) and infrared spectroscopy of the functionalized magnetic NTs demonstrated effective electrostatic binding (Supplementary Fig. 2g,h). Functionalized NTs were assembled with Exo^myo^ to work as ferromagnetic nanocarriers (NT-MAG-Exo^myo^) (Extended Data Fig. 1b). The amounts of nanoparticles and exosomes were chosen to maximize the loading efficiency while limiting exosome degradation and NT aggregation. In the best configuration, 500 μg of NT-MAG-Exo^myo^ was able to carry 5 × 10^9^ exosomes (Supplementary Fig. 2i–l). We analysed the stability of NT-MAG-Exo^myo^ in an aqueous environment by monitoring the release of Exo^myo^ at acidic and normal pH (6.5 and 7.4, respectively). The results indicated faster release of Exo^myo^ at pH 6.5 than at pH 7.4, with significant differences in the percentages of released Exo^myo^, especially in the first 6 h in a slightly acidic environment (Extended Data Fig. 1c), suggesting the possibility of modulating exosome release in a tissue pH-dependent^28–30^.

We investigated the response of NT-MAG-Exo^myo^ to the magnetic field in an aqueous environment by measuring the longitudinal (*R*_1_) and traverse (*R*_2_) magnetic relaxation rates as a function of the ferromagnetic NT concentration (Extended Data Fig. 1d). The *R*_2_ of NT-MAG-Exo^myo^ was enhanced by a factor of 2 with respect to NT-MAGs, probably due to better colloidal stability in the aqueous medium because of the presence of exosome. Nevertheless, we observed a stable and linear response to the external magnetic field for both *R*_1_ and *R*_2_. NT-MAG-Exo^myo^ had magnetic relaxivity as high as *R*_2_ = 10.7 s^−1^ (mg/ml)^−1^ and *R*_1_ = 0.7 s^−1^ (mg/ml)^−1^, resulting in an *R*_2_/*R*_1_ of 15.2, which makes the nanocarriers suitable as negative contrast agents for MRI (Extended Data Fig. 1d)^31^.

The controlled delivery of exosomes by an external magnetic field was assessed in vitro using a 3D-printed silicon/polylactic acid closed circuit with artificial blood medium (Extended Data Fig. 1e). The circuit, connected to a peristaltic pump for NT-MAG-Exo^myo^ circulation, featured one branch linked to an external reservoir via a septum covered by murine aortic wall; application of a 0.5 T magnetic field localized Exo^myo^ within the arterial elastic lamina, suggesting that NT-MAG-Exo^myo^ can effectively be guided through the blood vessels by magnetic force (Extended Data Fig. 1f and Supplementary Discussion 3).

**Fig. 4:**
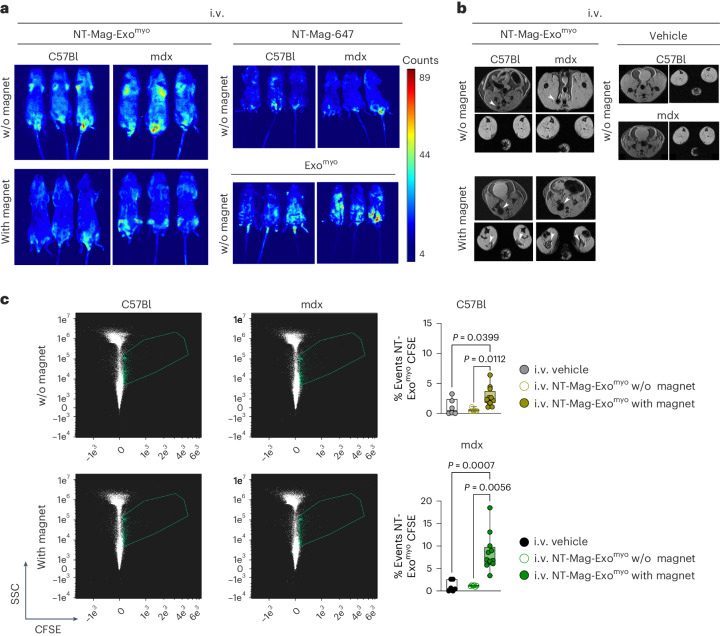
Short- and long-term in vivo biodistribution of NT-MAG-Exo^myo^. **a**,**b**, Optical bioluminescence (**a**) and MRI imaging (**b**) of 3-month-old C57Bl and mdx mice intravenously injected with NT-MAG-Exo^myo^ in the presence or absence of the magnet (*n* = 3 C57Bl and *n* = 3 mdx mice per condition) and analysed 24 h later. Intravenous injection of Exo^myo^ and NT-MAG-647 (*n* = 3 per animal model) without magnet placement were used as controls for bioluminescence. Intravenous injection of PBS (vehicle) was used as a control for MRI. The arrowheads in **b** indicate the predicted MRI signal of NT-MAG-Exo^myo^. **c**, Amnis ImageStream of NT-MAG-Exo^myo^ (left panel, in the scatter dot plot outlined areas) and quantification (right panel) of NT-MAG-Exo^myo^ in the hind limb muscles (quadriceps muscle, TA, gluteus, gastrocnemius) of 3-month-old C57Bl and mdx mice 30 days after intravenous injection of NT-MAG-Exo^myo^ (with or without magnet). Vehicle injection was used as control in both animal models; *n* = 3–6 mice per group in two independent experiments. Box plots show median, minimum to maximum values. C57Bl vehicle: median, 0.50; minimum, 0.00; maximum, 3.24; 25th percentile, 0.00; 75th percentile, 2.38; C57Bl Exo^myo^ without magnet: median, 0.43; minimum, 0.36; maximum, 1.10; 25th percentile, 0.37; 75th percentile, 0.66; C57Bl Exo^myo^ with magnet: median, 2.28; minimum, 1.05; maximum, 6.39; 25th percentile, 1.77; 75th percentile, 3.72; mdx vehicle: median, 0.54; minimum, 0.00; maximum, 2.62; 25th percentile, 0.00; 75th percentile, 2.62; mdx Exo^myo^ without magnet: median, 1.16, mininum, 0.85; maximum, 1.32; 25th percentile, 0.98; 75th percentile, 1.32; mdx Exo^myo^ with magnet: median, 6.86, minimum, 3.43; maximum, 18.51; 25th percentile, 6.14; 75th percentile, 18.51. Statistical analysis was performed using Kruskal–Wallis with Dunn’s multiple-comparison test. Source data

We tested the magnetic-field-assisted delivery of NT-MAG-Exo^myo^ in vivo using a ring-magnet-generated static field of 1.4 T. C57Bl and mdx mice were treated by systemic injection of Exo^myo^, NT-MAG-Alexa 647 or NT-MAG-Exo^myo^ in the presence or absence of the magnet and analysed 24 h later by optical bioluminescence imaging (Fig. 4a). The ring magnet was positioned around the limbs (Supplementary Fig. 3) during and for 30 min after injection to ensure the targeted delivery of NT-MAGs into the muscle. Without the external magnetic field, NT-MAG-Alexa-647-treated mice had a stronger signal near the proximal caudal vein with minimal to negligible signal in the whole body. In contrast, mice treated with Exo^myo^ and NT-MAG-Exo^myo^ had signals in various organs, including the muscles, liver, spleen, intestines and lungs. An amplified signal was observed in the mdx mice, probably due to impaired permeability of the dystrophic vasculature, resulting in fast clearance and vessel exit (Fig. 4a). We observed more confined tissue retention of NT-MAG-Alexa 647 than NT-MAG-Exo^myo^, probably due to the better colloidal stability of NT-MAGs when decorated with exosomes (Fig. 4a). NT-MAG-Exo^myo^-injected mice exposed to the magnetic field had a clear signal confined to the hind limbs (Fig. 4a). On MRI, signal localization at 24 h without magnet placement was around the proximal caudal vein in NT-MAG-Exo^myo^-injected C57Bl and mdx mice. A stronger muscle signal was observed in magnet-exposed hind limbs (Fig. 4b), confirming magnetic-field-driven localization of the exosomes in muscle after systemic administration of NT-MAG-Exo^myo^. Thirty days after intravenous NT-MAG-Exo^myo^ administration in C57Bl and mdx mice, significantly higher percentages of NT-MAG-Exo^myo^ were detected upon exposure to the magnetic field (Fig. 4c).

### Repetitive magnetic muscle targeting alleviates mdx myopathy

Systemic delivery of bare Exo^myo^ was inadequate to mitigate dystrophic muscle features, whereas repeated intramuscular delivery resulted in overall amelioration of the local muscle microenvironment and enhanced muscle function. However, the transferability to human patients would be constrained by the inability to treat all muscles in the body. To overcome this hurdle, we investigated the effects of weekly magnetic delivery of intravenously injected NT-MAG-Exo^myo^ in 3-month-old mdx and C57Bl mice (NT-MAG-Exo^myo^ I, one injection; NT-MAG-Exo^myo^ II, two injections; NT-MAG-Exo^myo^ III, three injections) compared to age-matched mdx and C57Bl mice that received three intravenous injections of PBS (vehicle), once a week. Compared to C57Bl mice, mdx mice injected with vehicle were not able to sustain exercise (Supplementary Fig. 4a). No differences in treadmill data were observed between NT-MAG-Exo^myo^ I, II and III mice and vehicle-administered C57Bl mice, indicating the lack of toxicity and side effects of NT-MAG-Exo^myo^ (Supplementary Fig. 4b). NT-MAG-Exo^myo^ III mdx mice were less prone to fatigue and displayed better treadmill performance independent of the work intensity (Supplementary Fig. 4c and Supplementary Discussion 4). NT-MAG-Exo^myo^ also alleviated myopathy in mdx mice, and the CSA increased with increasing number of NT-MAG-Exo^myo^ doses (Supplementary Fig. 5a and Supplementary Discussion 5). The frequency distribution confirmed the smaller CSA in mdx mice that received vehicle and NT-MAG-Exo^myo^ I (Supplementary Fig. 5a and Supplementary Discussion 5). Hydroxyproline quantification revealed that fibrosis was reduced in NT-MAG-Exo^myo^-treated mdx mice compared with controls, with a sharp drop in muscles from NT-MAG-Exo^myo^ III mice (Supplementary Fig. 5b).

**Fig. 5:**
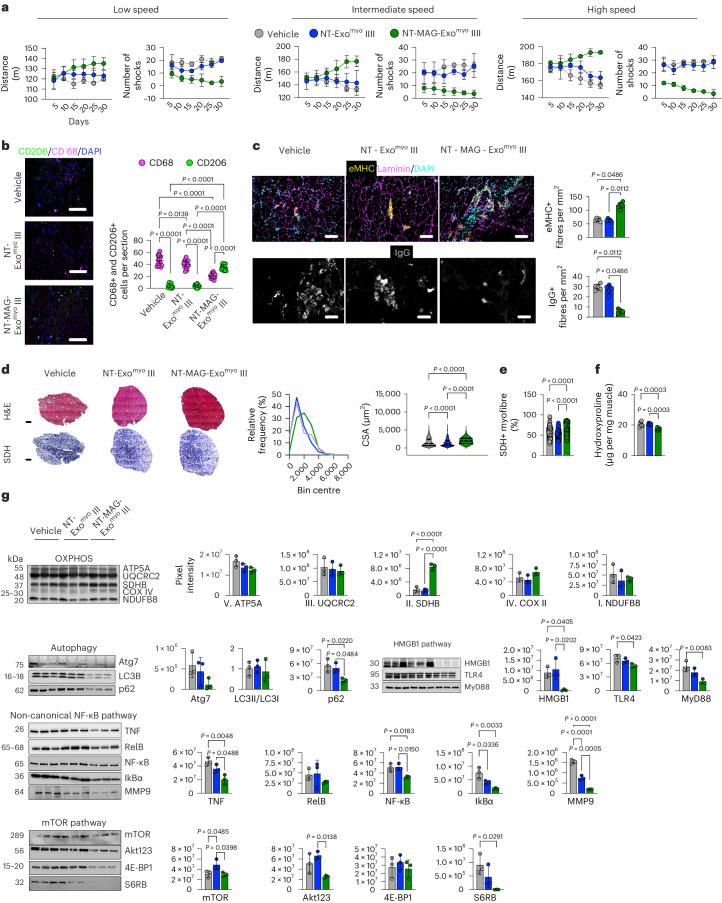
Magnetic delivery of NT-MAG-Exo^myo^ improves muscle function in mdx mice. Three-month-old mdx mice were intravenously injected with three doses, once per week, of NT-MAG-Exo^myo^ (NT-MAG-Exo^myo^ III) delivered through magnet positioning around murine muscles (*n* = 8 mdx mice). Mice intravenously injected with three doses of non-magnetic NT-Exo^myo^ (NT-Exo^myo^ III) delivered through magnet positioning and mice receiving three intravenous injections of vehicle (that is, PBS) once a week were used as controls (*n* = 8 mdx mice per group). All mice were killed 30 days after the first injection. **a**, Treadmill performance of mice injected with NT-MAG-Exo^myo^ III, NT-Exo^myo^ III and vehicle. Data were recorded at 20 cm s^−1^ (low intensity), 25 cm s^−1^ (intermediate intensity) and 30 cm s^−1^ (high intensity). Results from the first injection to the day on which the mice were killed are expressed as average distance and number of shocks ± s.d.; two-way ANOVA followed by post hoc Tukey multiple-comparison test. *n* = 3 mice per group. *P < 0.05, **P < 0.01, ***P < 0.001 and ****P < 0.0001 for comparison between NT-MAG-Exo^myo^ III and NT-Exo^myo^ III; exact P values are reported for vehicle compared to NT-MAG-Exo^myo^ III-administered mice. **b**, Immunofluorescent staining of NT-MAG-Exo^myo^ III- and non-magnetic NT-Exo^myo^ III-injected muscle tissue. Infiltration of proinflammatory and anti-inflammatory macrophages was detected and quantified using CD68 (magenta) and CD206 (green) immunoreactivity, respectively. Scale bars, 50 μm. Immunoreactivity was quantified by ImageJ software. Data are presented as mean ± s.d.; *n* = 3 mice per group, *n* = *5* slides per mouse. Statistical analyses were performed using one-way ANOVA followed by post hoc Tukey multiple-comparison test. **c**, Regenerating and necrotic myofibres in NT-MAG-Exo^myo^ III- and non-magnetic NT-Exo^myo^ III-injected muscle tissue were stained with embryonic myosin (eMHC, yellow) and laminin (magenta), or with goat anti-mouse IgG (grey). Scale bars, 50 μm. Immunoreactive fibres were quantified per mm^2^ using ImageJ software; *n* = 5 mice per group. Data are presented as mean ± s.d. Statistical analyses were performed using Kruskal–Wallis with Dunn’s multiple-comparison test. **d**, Representative images of muscle tissue stained with H&E with quantification of the myofibre cross-sectional area. Scale bars for H&E, 200 μm. For each group, H&E images were counted from *n* = 4 mice: *n* = 3,287 myofibres for vehicle-treated mdx (median, 1,200.087 μm^2^; 25th percentile, 1,200.08 μm^2^; 75th percentile, 2,420.57 μm^2^), *n* = 4,553 myofibres (median, 1,144.176 μm^2^; 25th percentile, 639.52 μm^2^; 75th percentile, 2,013.52 μm^2^) for non-magnetic NT-MAG-Exo^myo^ III; *n* = 3,294 myofibres (median, 1,947.214 μm^2^; 25th percentile, 1,260.30 μm^2^; 75% percentile, 2,682.62 μm^2^) for NT-MAG-Exo^myo^ III. Violin plots showing CSA median (dotted lines) and quartiles (solid lines); Kruskal–Wallis with Dunn’s multiple-comparison test. **e**, Representative SDH staining and quantification of the percentage of SDH+ myofibres. *n* = 5 mice per group; *n* = 24–26 slices per mouse. Data are presented as mean ± s.d.; Kruskal–Wallis with Dunn’s multiple-comparison test. **f**, Fibrosis was quantified using the hydroxyproline assay and muscle tissue isolated from NT-MAG-Exo^myo^ III- and non-magnetic NT-Exo^myo^ III-injected mice. *n* = 3 mice per group in two independent experiments; data are presented as mean ± s.d. Statistical analysis was performed using one-way ANOVA followed by post hoc Tukey multiple-comparison test. **g**. Cropped representative images of the Western blot showing the expression of proteins involved in OXPHOS complexes, autophagy mechanisms and inflammation (HMGB, non-canonical NF-κB and mTOR pathways) in muscle tissue from mdx mice treated with PBS (Vehicle), non-magnetic NT-MAG-Exo^myo^ III and NT-MAG-Exo^myo^ III. Bands were normalized for total protein loading (visualized by stain-free technology, in the Chemidoc system). Data are presented as mean ± s.d.; *n* = 3 mice per group; one-way ANOVA followed by post hoc Tukey multiple-comparison test. Source data

Furthermore, to investigate whether improved muscle performance in mdx mice was specifically ascribable to Exo^myo^ accumulation in muscles, driven by a magnetic field, we compared the effects of weekly magnetic delivery of intravenously injected NT-MAG-Exo^myo^ (NT-MAG-Exo^myo^ III, three injections) and NT-Exo^myo^ (NT-Exo^myo^ III, three injections), non-responsive to magnetic field, in 3-month-old mdx mice. NT-MAG-Exo^myo^ III mdx mice presented with reduced muscle exhaustion and improved endurance compared with NT-Exo^myo^ III mdx mice, which presented a fatigue pattern comparable to the vehicle-treated mdx group (Fig. 5a and Supplementary Discussion 6).

The number of Ly6C+ infiltrating macrophages was decreased in TA muscle from NT-MAG-Exo^myo^ III mdx mice compared with vehicle-treated and NT-Exo^Myo^ III mdx mice (Supplementary Fig. 6). The NT-MAG-Exo^myo^ III mdx mice had a notable decrease in the number of M1 CD68+ CD206− macrophages, accompanied by a concurrent increase in M2 CD68+ CD206+ macrophages (Fig. 5b), suggesting that NT-MAG-Exo^myo^ facilitated M2 polarization, indicating a potential immunomodulatory effect. Histological analyses of TA muscles isolated from NT-MAG-Exo^myo^ III mdx mice showed significantly less muscle degeneration than in vehicle-treated and NT-Exo^myo^ III mdx mice, as indicated by a reduced number of degenerating fibres (IgG+) and an increased percentage of healthy peripherally nucleated fibres and regenerating eMHC+ fibres (Fig. 5c). In addition, myofibre size was significantly increased in TA from NT-MAG-Exo^myo^ III mdx mice compared with vehicle-treated and NT-Exo^myo^ III mdx mice, suggesting metabolic reprogramming in the muscle (Fig. 5d). Accordingly, the number of SDH+ myofibres increased in the TA from NT-MAG-Exo^myo^ III mdx mice (Fig. 5e). Hydroxyproline was significantly decreased in TA from NT-MAG-Exo^myo^ III mdx mice compared with vehicle-treated and NT-Exo^myo^ III mdx mice, confirming reduced fibrosis (Fig. 5f).

To clarify the modifications to muscle metabolism, we studied the effect of NT-MAG-Exo^myo^ III on oxidative phosphorylation (OXPHOS) proteins and related systems. No changes were observed in the muscle expression of different subunits of respiratory complex I, III, IV and V between the NT-MAG-Exo^myo^ III and NT-Exo^myo^ III mdx groups (Fig. 5g). However, the complex II (SDH) subunit was significantly overexpressed in NT-MAG-Exo^myo^ III mdx mice (Fig. 5g). Mdx muscles exhibit altered mitochondrial respiration^32^, and heightened high mobility group box 1 (HMGB1) release from necrotic fibres and M1 macrophages^33–35^. We found that HMGB1 expression was significantly lower in muscles from NT-MAG-Exo^myo^ III mdx mice compared with both vehicle and NT-Exo^myo^ III mdx mice (Fig. 5g). Notably, downstream HMGB1 receptors were significantly downregulated in muscles from NT-MAG-Exo^myo^ III mdx mice. To investigate whether the reduction in the HMGB1 pathway was associated with decreased inflammatory pathways, we specifically focused on the proinflammatory TNF and NF-κB signalling pathways. Downregulation of TNF and similar amounts of IkBα were observed in muscles from NT-MAG-Exo^myo^ III mdx mice (Fig. 5g). Compared with both vehicle-treated and NT-Exo^myo^ III, the NT-MAG-Exo^myo^ III mdx mice had reduced levels of total NF-κB and NF-κB subunit RELB (Fig. 5g). Extracellular HMGB1 serves as a promoter of autophagy through the mTOR mechanisms^36^, and dystrophic muscles in the NT-MAG-Exo^myo^ III group showed a significant reduction in the AKT/mTOR/4E-BP12/S6Rb signalling pathway. This group also tended to have reduced levels of autophagy-selective substrates (Fig. 5g).

### Magnetic delivery of Exo^myo^ shifts dystrophic muscle traits

Next, we further characterized the molecular traits induced by repetitive intravenous infusion of NT-MAG-Exo^myo^ (NT-MAG-Exo^myo^ I, II and III; Supplementary Information, RNA-seq file 1) compared to vehicle-administered mice, and by NT-MAG-Exo^myo^ III compared to non-magnetic NT-Exo^myo^ injection (NT-Exo^myo^ III; Supplementary Information, RNA-seq file 2). Thirty days after the first injection, the mice were killed, and hind limb muscles were prepared for RNA sequencing analyses of single muscle fibres and muscle-infiltrating macrophages containing CFSE-positive exosomes (Ly6C^hi^CFSE^hi^; iMACs).

**Fig. 6:**
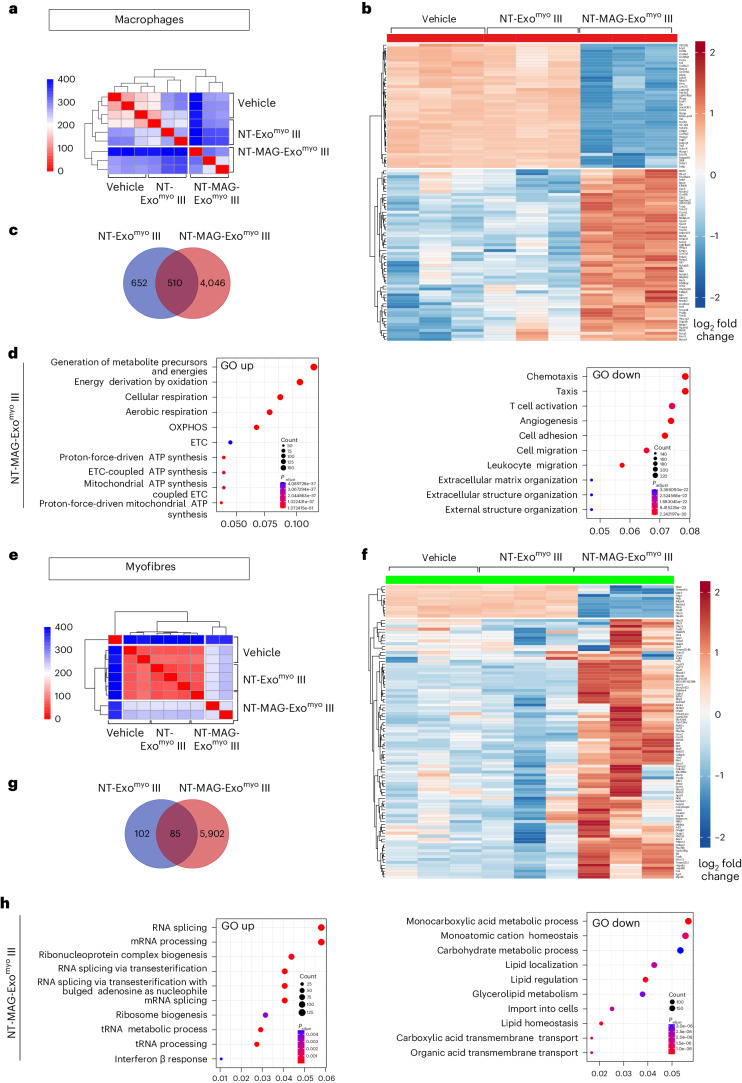
Magnetic delivery of NT-MAG-Exo^myo^ determines anti-inflammatory polarization of muscle-infiltrating macrophages and muscle reprogramming in mdx mice. Transcriptomic analysis of freshly FACS-purified circulating macrophages that infiltrated hind limb muscle tissues and entrapped CFSE+ exosomes (Ly6C^hi^CFSE^hi^; iMAC) in non-magnetic NT-Exo^myo^ III (*n* = 3) and magnetic NT-MAG-Exo^myo^ III (*n* = 3) mice (upper panel, **a**–**d**). Transcriptomic analysis of single muscle fibres (MS) from the hind limb muscle tissues from non-magnetic NT-Exo^myo^ III (*n* = 3), and magnetic NT-MAG-Exo^myo^ III (*n* = 3) mice (bottom panel, **e**–**h**). Mdx mice systemically injected with PBS were used as controls (vehicle, *n* = 3). **a**,**e**, Heatmap of Euclidean distances between log_2_ counts per million gene expression values across all macrophage samples (**a**) and myofibres (**e**). The colour scale ranges from 0 (indicating identical gene expression) in red to 400 (indicating the highest distance) in blue. **b**,**f**, Heatmap of log CPM expression values for the top 100 differentially expressed genes (DEGs) between groups, for muscle infiltrating macrophages (**b**) and myofibres (**f**). The colour scale ranges from low expression (blue) to high expression (red). Expression values were standardized. **c**,**g**, Venn diagram of the shared DEGs between groups, for muscle infiltrating macrophages (**c**) and myofibres (**g**). **d**,**h**, GO pathway analysis of upregulated (left) and downregulated (right) genes in muscle infiltrating macrophages (**d**) and myofibres (**h**). A one-sided version of Fisher’s exact test was used to determine whether known biological functions or processes are overrepresented in the list of differentially expressed genes. The *P* values are adjusted according to the Benjamini–Hochberg method. *P* value and *q* value cut-offs are 0.01 and 0.05, respectively. The size of each dot is proportional to the number of genes in the corresponding category. The colour scale represents the significance of the analysis for each category: red denotes higher significance; blue denotes lower significance.

**Extended Data Fig. 2:**
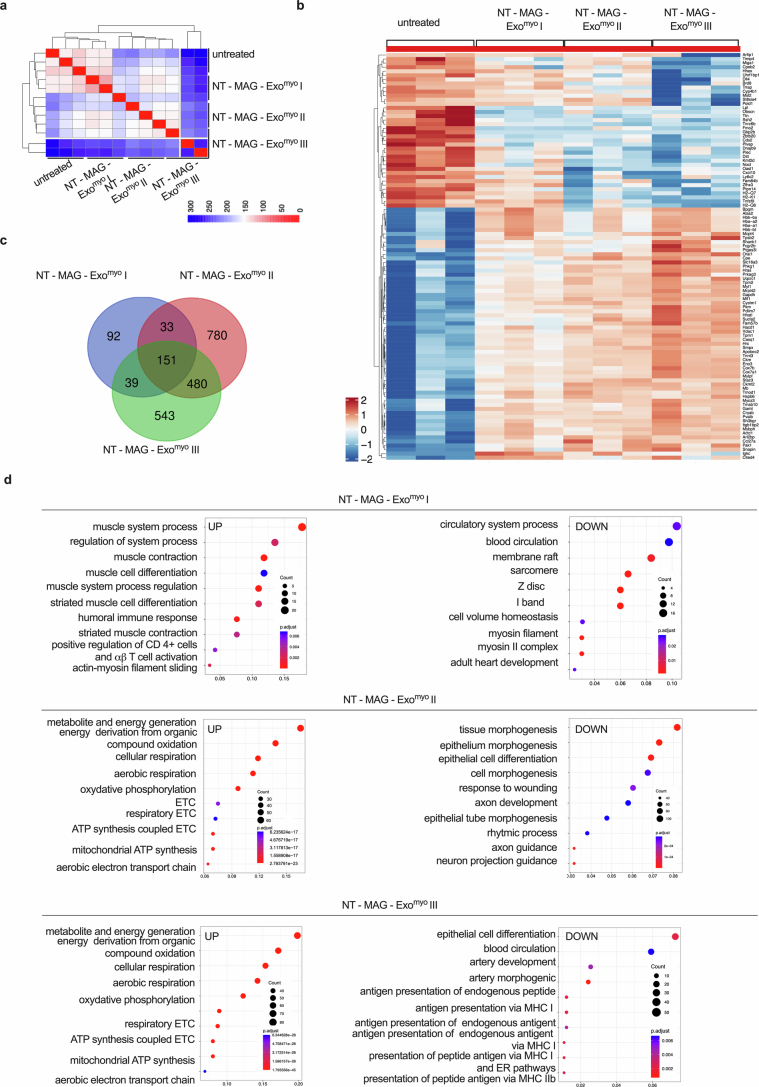
Transcriptomic profiling of muscle iMAC isolated from mdx mice treated with NT-MAG-Exomyo III. Transcriptomic analysis of freshly FACS-purified circulating macrophages that infiltrate hind limb muscle tissues and entrapped CFSE+ exosomes (Ly6ChiCFSEhi; iMAC) in NT-MAG-Exomyo I (n = 3), NT-MAG-Exomyo II (n = 3) and NT-MAG-Exomyo III (n = 3). Mdx mice systemically injected with PBS was used as control (vehicle, n = 3). **a**, Heatmap representation of Euclidean distances between log2 counts per million gene expression values across all samples. The colour scale represents the distance values, ranging from 0 (indicating identical gene expression) in red to 300 (indicating the highest distance) in blue. The heatmap provides a visual representation of the similarity or dissimilarity of gene expression patterns among samples. **b**, Heatmap representation of log CPM expression values for the top 100 differentially expressed genes between groups. The colour scale ranges from blue (indicating low expression) to red (indicating high expression). Expression values have been standardized. **c**, The Venn diagram representation of the shared DEGs between groups. **d**, Gene Ontology (GO) pathway analysis of up- and downregulated genes. One-sided version of Fisher’s exact test used to determine whether known biological functions or processes are overrepresented in the list of differentially expressed genes. The p-values are adjusted according to Benjamini–Hochberg method. P-value and q-value cutoff are 0.01 and 0.05, respectively. The size of each dot is proportional to the number of genes in the corresponding category. The colour scale represents the significance of the analysis for each category: red denotes higher significance; blue denotes lower significance.

**Extended Data Fig. 3:**
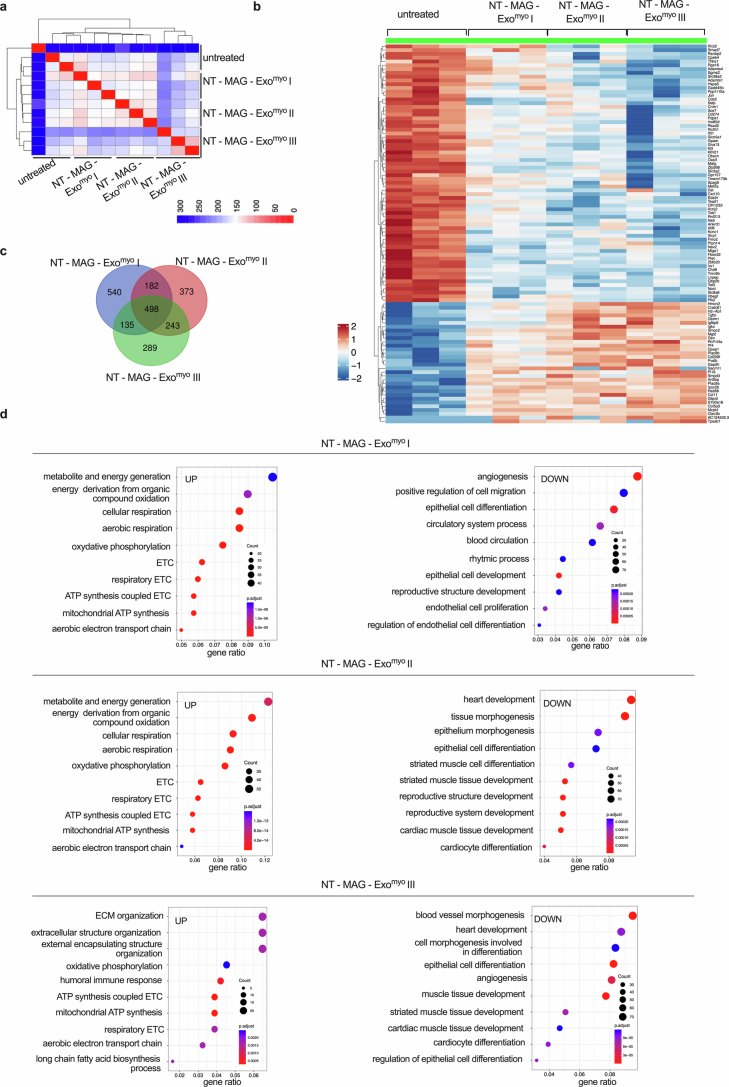
Transcriptomic profiling of muscle fibres isolated from mdx mice treated with NT-MAG-Exomyo III. Transcriptomic analysis of single muscle fibres (MS) of hind limb muscle tissues of mdx vehicle (n = 3), NT-MAG-Exomyo I (n = 3), NT-MAG-Exomyo II (n = 3) and NT-MAG-Exomyo III. **a**, Heatmap representation of Euclidean distances between log2 counts per million gene expression values across all samples. The colour scale represents the distance values, ranging from 0 (indicating identical gene expression) in red to 300 (indicating the highest distance) in blue. The heatmap provides a visual representation of the similarity or dissimilarity of gene expression patterns among samples. **b**, Heatmap representation of log CPM expression values for the top 100 differentially expressed genes between groups. The colour scale ranges from blue (indicating low expression) to red (indicating high expression). Expression values have been standardized. **c**, The Venn diagram representation of the shared DEGs between groups. **d**, Gene Ontology (GO) pathway analysis of up- and downregulated genes. One-sided version of Fisher’s exact test used to determine whether known biological functions or processes are overrepresented in the list of differentially expressed genes. The p-values are adjusted according to Benjamini–Hochberg method. P-value and q-value cutoff are 0.01 and 0.05, respectively. The size of each dot is proportional to the number of genes in the corresponding category. The colour scale represents the significance of the analysis for each category: red denotes higher significance; blue denotes lower significance.

Substantial dissimilarities in the gene expression patterns were found between iMACs and single muscle fibres from NT-MAG-Exo^myo^ groups compared to controls (Extended Data Fig. 2a–c, Extended Data Fig. 3a,b and Supplementary Discussion 7), and NT-MAG-Exo^myo^ III compared to NT-Exo^myo^ III (Fig. 6a–c,e–g and Supplementary Discussion 7). Gene Ontology (GO) pathway analysis revealed that the top 15 upregulated pathways in iMACs (Fig. 6d) shared among the three NT-MAG-Exo^myo^ groups were associated with ‘mitochondrial,’ ‘aerobic respiration’ and ‘oxidative phosphorylation’ (Extended Data Fig. 2d and Supplementary Discussion 7). Gene set enrichment analysis (GSEA) and volcano plot analysis of the NT-MAG-Exo^myo^ III group showed upregulation of oxidative genes and a shift in iMAC phenotype towards anti-inflammatory pathways (Supplementary Fig. 7 and Supplementary Discussion 7). GSEA and volcano plot analysis of iMACs from the comparison of NT-MAG-Exo^myo^ III versus NT-Exo^myo^ III confirmed the upregulation of genes involved in reprogramming metabolism and mRNA translation of M2 macrophages in NT-MAG-Exo^myo^ III, whereas a limited pool of dysregulated genes was found in NT-Exo^myo^ III versus vehicle (Supplementary Figs. 8a and 9a and Supplementary Discussion 7). KEGG analysis of iMACs from NT-Exo^myo^ III revealed upregulated genes in inflammatory/apoptosis signalling pathways (‘prion disease’, ‘Huntington disease’, ‘diabetic cardiomyopathy’) (Supplementary Fig. 9a). In addition, NT-Exo^myo^ III had repressed genes in pathways that are known to be induced by canonical M2 activation^37^ (‘tight junction’, ‘adherens junction’) (Supplementary Fig. 9a). Taken together, these data demonstrate that Exo^myo^ uptake by iMACs shifts their phenotype toward a profile characterized by enhanced oxidative and anti-inflammatory M2 metabolic pathways.

The oxidative pathway was also upregulated in single muscle fibres isolated from NT-MAG-Exo^myo^ II and NT-MAG-Exo^myo^ III mice (Extended Data Fig. 3d and Supplementary Discussion 8). GSEA of myofibres from NT-MAG-Exo^myo^ II and NT-MAG-Exo^myo^ III mice revealed enrichment of genes involved in skeletal muscle contraction and transition between fast and slow fibres (Supplementary Fig. 10 and Supplementary Discussion 8). Upregulated KEGG pathways confirmed enrichment of ‘oxidative phosphorylation’ and ‘cardiac muscle contraction’ genes in single muscle fibres from NT-MAG-Exo^myo^ mice, regardless of dose (Supplementary 10). Single muscle fibres from NT-MAG Exo^myo^ III mice showed upregulated GO pathways associated with RNA splicing/maturation and downregulation of several genes involved in the extracellular matrix response to inflammatory stimuli compared with the NT-Exo^myo^ group (Fig. 6h, Supplementary Figs. 8b and 9b and Supplementary Discussion 8). The average gene expression in single muscle fibres from NT-Exo^myo^ III mice recapitulated the main features of the vehicle group (Supplementary Fig. 9b). The above results demonstrate a metabolic reprogramming of TA muscle fibres after magnetic delivery of NT-MAG-Exo^myo^.

## Discussion

Significant advances in the field of exosome biology have created new possibilities for therapeutic interventions^38,39^. Exosomes can be engineered to deliver therapeutic payloads, with the ability to target specific cells or tissues^5,40–42^. Given the growing interest in exosomes promoting muscle regeneration^3,4,6^, we investigated the efficacy of magnetic delivery in a well-defined model of DMD. To modulate the inflammatory milieu of dystrophic skeletal muscle, we enriched exosomes for ANXA1, a proresolving mediator in anti-inflammatory and repair mechanisms^18,43,44^. In vitro, enriched exosomes effectively promoted the transition of proinflammatory macrophages to an anti-inflammatory state and facilitated the myogenic maturation of dystrophic muscle progenitors. In vivo, we exploited ferromagnetic nanocarriers for controlled delivery and muscle accumulation of Exo^myo^ upon systemic injection. With repeated weekly administration, effective muscle repair was observed in mdx mice. The uptake of exosomes by infiltrating macrophages suppressed inflammatory pathways and caused a metabolic shift towards enhanced oxidative metabolism in dystrophic muscle fibres. This muscle fibre–macrophage interaction reduces muscle fibre remodelling, thereby improving muscle contraction. The proposed nanocarrier-based approach is an affordable, efficient and low-cost strategy for developing therapeutic exosomes that could improve the quality of life for individuals with muscular dystrophies.

## Methods

### Animals and ethics statement

Animal experiments conformed with Italian law (DLvo 116/92) and were approved by the Animal Ethics Committee of the Department of Molecular Biotechnology and Health Sciences of Turin and the Ministry of Health (PR 72833) and Istituto Besta of Milan (833/2019-PR). Two-month-old male C57Bl/10 and mdx mice from Charles River were housed in ventilated cages with a 12 h light/dark cycle and free access to water and standard autoclaved chow. Mice were allowed to acclimate for at least 4 weeks prior to analysis. For all experiments, we used 3-month-old male C57Bl and mdx mice.

For intramuscular injection, mice were injected in the TA muscle. For systemic administration, mice were injected in the tail vein. A dose of 10^9^ Exo^myo^ was injected in 100 μl PBS 1×. Mice were anaesthetized with 2% isoflurane before being killed by cervical dislocation.

In one experimental group, mdx mice were intramuscularly (*n* = 36) or intravenously (*n* = 36) injected with Exo^C2C12^ or Exo^myo^ and analysed 24 h, 7 days and 21 days after injection (*n* = 6 per time point). Untreated mdx mice (*n* = 6 each time point) were used as controls.

In another experimental group, mdx mice were intramuscularly (*n* = 24) or intravenously (*n* = 18) injected with Exo^myo^ once, twice or three times, once per week (*n* = 6 mice for systemic administration, *n* = 12 mice for TA injections). Untreated mdx (*n* = 6) were used as controls. Mice were killed 30 days after the first injection.

For optical imaging and MRI of the biodistribution at 24 h, C57Bl mice (*n* = 3 each group) and mdx mice (*n* = 3 each group) were injected intravenously with Exo^myo^ or NT-MAG-647 or NT-MAG-Exo^myo^. NT-MAG-Exo^myo^ injection was evaluated with or without magnet placement. PBS-injected mice (*n* = 3 C57Bl, *n* = 3 mdx) served as controls.

For testing the magnetic-field-assisted delivery of NT-MAGs in vivo, we used a ring-magnet-generated static field of 1.4 T. Upon systemic injection, the ring magnet was positioned around the mouse limbs and maintained in place for 30 min after treatment.

For Amnis imaging flow cytometry analysis of long-term retention of NT-MAG-Exo^myo^ within muscles, 30 days after systemic injection, C57Bl and mdx mice were injected with PBS (*n* = 3 each) or NT-MAG-Exo^myo^ (*n* = 6 with magnet placement; *n* = 3 without magnet placement).

To evaluate the effects of weekly magnetic delivery NT-MAG-Exo^myo^, we intravenously injected 3-month-old mdx mice once (NT-MAG-Exo^myo^ I, *n* = 8), twice (NT-MAG-Exo^myo^ II, *n* = 8) or three times (NT-MAG-Exo^myo^ III, *n* = 8) once a week. As controls, one group of mdx mice was administered three injections of non-magnetic NT-Exo^myo^ (NT-Exo^myo^ III, *n* = 8), and a second group received three intravenous injections of PBS (vehicle, *n* = 8), once a week. For motor behaviour analysis and to exclude the possibility of NT-MAG-Exo^myo^ toxic systemic effects, 3-month-old C57Bl mice were also intravenously injected with NT-MAG-Exo^myo^, once (NT-MAG-Exo^myo^ I, *n* = 3), twice (NT-MAG-Exo^myo^ II, *n* = 3) or three times (NT-MAG-Exo^myo^ III, *n* = 3) once a week, or with three intravenous injections of PBS (vehicle, *n* = 3). Mice were killed 30 days after the first injection.

### Exosome isolation and characterization

Murine C2C12 myoblast cells (CRL-1772, ATCC) were cultured in DMEM high glucose until 60% confluent. The medium was changed to exosome-depleted 10% FBS-supplemented DMEM medium for 48 h and cell culture media was collected and centrifuged to remove debris. The supernatant was filtered and sequentially ultracentrifuged for 2 h at 4 °C (40,000*g* and 130,000*g*; Optima XE, Ti 45 fixed-angle rotor) to pellet exosomes. Protein concentrations were measured using the microBCA Protein Assay Kit. For TEM, the pellet was resuspended in PBS 1× and 5 µl directly loaded onto formvar carbon-coated grids^45,46^. Exosome concentration and size distribution were determined by NTA using a NanoSight NS300. The supernatant was collected from C2C12 cells pretreated with 100 μM cytochalasin D to isolate ANXA1-primed exosomes. For Western blotting, 10^6^, 10^7^, 10^8^ and 10^9^ exosomes (15 µl) were lysed in RIPA assay buffer (25 mM Tris (pH 7.5), 150 mM NaCl, 0.1% SDS, 0.5% sodium deoxycholate, 1% Triton X-100). Exosome lysates were diluted in Laemmli sample buffer 4× and boiled at 95 °C for 10 min; for CD9, CD81 and CD63 Western blot, exosome lysates were diluted in non-reducing LDS sample buffer 4× (ref. ^47^). Antibodies were incubated with the membrane overnight at 4 °C in 5% milk in TBS-T with shaking. The dilution of antibodies is listed in Supplementary Table 1. Densitometric analysis was performed using ImageJ (http://rsbweb.nih.gov/ij/).

### Proteomic analysis

Exosome pellets (10^10^ Exo^myo^ and 10^10^ Exo^C2C12^) were suspended in 0.1 M ammonium bicarbonate (pH 7.9) and sonicated with a Branson Sonifier^48^. Exosomal proteins (50 ± 0.5 µg) were analysed by nanochromatography on a cHiPLC-nanoflex system coupled to a Q-Exactive mass spectrometer using a 65 min gradient of 5–45% eluent B (A, 0.1% formic acid in water; B, 0.1% formic acid in acetonitrile) at a flow rate of 300 nl min^−1^. Full mass spectra were recorded in positive-ion mode over 400–1,600 *m*/*z* at 70,000 full-width at half-maximum (FWHM) resolution, followed by 10 MS/MS spectra at a resolution of 17,500 FWHM. All generated data were searched using Proteome Discoverer 2.1 software (Thermo Fisher Scientific) based on the SEQUEST search engine and *Mus musculus* UniProt FASTA database (UP000000589, March 2018, 61,307 entries; www.uniprot.gov). The false discovery rate was set to 1, meaning 100%, to allow for subsequent peptide-spectrum match (PSM) rescoring via Prosit and Percolator implemented in Proteomics DB^49^. After rescoring, PSMs and peptides were filtered at a false discovery rate of 1% (calculated at sample level). The MS data have been deposited with the ProteomeXchange Consortium via the PRIDE^50^ partner repository with the dataset identifier ftp://MSV000094590@massive.ucsd.edu.

### Exosome CFSE labelling

For CFSE staining, exosomes were labelled with 40 μM CFSE dye for 60 min at 4 °C. To eliminate unbound fluorophore, we concentrated exosomes using a 3 kDA MWKO filter for 30 min at 15,000*g* at 4 °C. CFSE-labelled samples were retained above the filter and collected for further analysis. Unless otherwise specified, Exo^myo^ were labelled with CFSE for immunofluorescence characterization and in vivo detection.

### In vitro model of Exo^myo^ and Exo^C2C12^ treatment

To induce myogenic differentiation, 70% confluent fluorescence-activated cell sorting (FACS)-isolated SCs were grown in exosome-depleted low-serum fusion-promoting medium (high-glucose DMEM with 2% FBS) with or without the addition of 10^8^ Exo^myo^ and 10^8^ Exo^C2C12^. Myotube formation was evaluated after 7 days of differentiation. Nuclei and cytoskeletal staining were carried out with desmin and 1 μM 4,6-diamidino-2-phenylindole (DAPI). Images were acquired using a Leica DMIRE2 microscope, and the fusion index was determined for each picture by dividing the total number of nuclei in myotubes by the total number of counted nuclei. Myotube area, length and width were extracted from each image using ImageJ. To characterize polarization, freshly isolated macrophages were treated for 24 h in the presence or absence of 10^8^ Exo^myo^ and 10^8^ Exo^C2C12^ in DMEM High Glucose, 20% exosome-depleted FBS, 30% L929 cell (ATCC) conditioned medium containing 1% amphotericin B and 100 μg ml^−1^ penicillin/streptomycin (Supplementary Table 2).

### Immunocapture of Exo^Myo^

TA muscles were collected from mdx mice intramuscularly injected with Exo^myo^. The tissues were dissociated into single-cell suspensions in 0.2 mg ml^−1^ liberase, and mononuclear cells collected after layering onto Optiprep gradient. CD45+ cells were purified by positive selection using an MACS separation MS column and a MiniMACS separator according to the manufacturer’s instructions. Negatively selected CD45− cells were eluted through the LS column and centrifuged at 500*g* for 5 min at 4 °C. The magnetically labelled and retained CD45+ cells were flushed from the column, collected and centrifuged at 500*g* for 5 min at 4 °C. The CD45− fraction was labelled for myogenic marker ITGA 7 PE viol 770 and the CD45+ fraction for macrophage marker CD68 APC. For the same samples, CFSE-positive cells were acquired using the BD Canto II machine and analysed by FlowJo 9 software (at least 5–10 × 10^4^ events for each gate). For exosome collection from murine muscles, TA and quadriceps muscles were harvested from mice injected intramuscularly or intravenously with Exo^myo^ and Exo^C2C12^. The tissues were cut into 1 mm^3^ pieces and digested in 2 mg ml^−1^ collagenase D in high-glucose DMEM for 1 h at 37 °C with light shaking. After digestion, the solution was sequentially filtered through 100 μm, 40 μm and 1 μm filters. Exosomes were captured using an ExoFlow 2.0 CD63 kit for tissue culture media according to the manufacturer’s instructions and a commercial magnetic stand, DynaMag-2. After washing away bead-bound exosomes and excess stain, the expression of CFSE and exosomal markers CD9 and CD81 was confirmed by FACS analysis. Anti-CD9-APC Cy7 and anti-CD81-PE Cy7 were incubated with exosomes for 20 min at 4 °C. Controls were incubated with isotype control mouse IgG1k-PE antibody. The dilutions of antibodies are listed in Supplementary Table 1. Samples were analysed on a BD Canto II machine and quantified using FlowJo 9. For Amnis analysis of NT-MAG-Exo^myo^, filtered hind-limb-muscle-derived supernatants were run directly on an ImageStream^X^ MK II.

### Synthesis of stoichiometric Mg_3_(Si_2_O_5_)(OH)_4_ NTs

NTs were synthesized by an optimized method^51^. A reactor with a 100 cm^3^ polypropylene vessel was used for the hydrothermal reaction (250 °C, 16 h) of 1,522 mg Na_2_SiO_3_ and 764 mg MgCl_2_ in 220 ml of an aqueous solution of NaOH (0.4 M) (Supplementary Table 2). The solution precipitate was washed repeatedly with deionized water and dried (3 h, 110 °C).

### NT functionalization with Alexa Fluor 647 or Exo^myo^

First, 40 mg chrysotile powder was suspended in 10 ml of 5 × 10^−3^ PBS (pH 7.4), and then 2.5 ml of Alexa Fluor 647 solution (9.8 × 10^−6^ M in PBS) was added slowly while stirring^52^. NTs were centrifuged and dried at 50 °C for 3 h. Different degrees of surface coverage were obtained using 1:5 and 1:10 dilutions of the PBS Alexa Fluor 647 solution. The optical properties of stained NTs were checked by measuring the steady-state photoluminescence with a Cary Eclipse spectrofluorometer. Time-resolved photoluminescence experiments were performed with an Edinburgh Instruments FLS-980 spectrofluorometer. To attach Exo^myo^, NTs were suspended in PBS 1×, and Exo^myo^ were added at various dilutions (1:10–1:100 v/v) overnight. Adsorption occurred at 4 °C for 12 h on a rotating shaker.

### Structural and compositional characterization

Powder X-ray diffraction was performed with a Miniflex 600 diffractometer in Bragg–Brentano configuration. Attenuated total reflection Fourier-transform infrared spectra were obtained on dried samples with a Nicolet iS20 spectrometer.

### High-resolution TEM

TEM observations were carried out using a JEM 2100 Plus electron microscope. First, 1–2 mg of powder was dissolved in 2 ml of distilled water, and then 3 μl of the solution was placed on carbon-coated copper grids.

### Exo^myo^ binding and adsorption onto fluorescent NTs

First, 10^9^ Exo^myo^ were added directly to PBS containing sonicated Alexa 647-labelled NTs at various dilutions (1:10–1:100, v/v). Adsorption then occurred as described above. For dSTORM high-resolution fluorescence imaging using a Leica SR GSD 3D TIRF, samples were deposited on a coverslip previously coated with a Cell Tak-compatible adhesive.

### ImageStream acquisition and analysis

Exo^myo^, Alexa 647-labelled NTs (50 µl) and Exo^myo^ absorbed onto Alexa 647-labelled NTs were suspended in 100 µl 0.1 µm filtered PBS 1× for imaging flow cytometer (ImageStream^X^ MK II System, Amnis). The ImageStream^X^ MK II System was equipped with three lasers (405, 488 and 642 nm), a six-channel charge-coupled device camera and the Multimag option, but no extended depth-of-field option. All events were collected with a 60× (numerical aperture, 0.9) objective at low speed and high sensitivity with 7 μm core size. Speed beads were running throughout the acquisition. Exo^myo^ CFSE was excited by a 488 nm laser and emission collected in Ch02. AlexaFluor647 was excited by a 642 nm laser and emission collected in Ch05. Single-stained samples were acquired with identical laser settings. Side-scatter illumination was used for compensation. Purified Exo^myo^ were diluted 1:100 to avoid swarming effects and images analysed using IDEAS 6.2 software: Exo^Myo^ were identified by the signal intensity in Ch02 (intensity_Ch02) plotted against intensity_Ch06 as events with very low scatter and low/mid green fluorescence intensity. To avoid false positives, CFSE dye alone was also analysed. NT-Alexa 647 was identified by intensity_Ch05 and their strong scattering signal in Ch06. Exo^myo^-loaded NT-Alexa 647 was identified by plotting intensity_Ch05 against intensity_Ch02.

For Amnis analysis of NT-MAG- Exo^myo^, supernatants derived from hind limb muscles were prepared as described above and run on an ImageStream^X^ MK II. To obtain a collection gate (R6) that can confirm the presence of NT-MAG-Exo^myo^ in the samples, a scatter plot was drawn based on the scattering properties of the fibres (intensity_Ch06) and the CFSE signal of the exosomes (intensity_Ch02).

### Synthesis of Fe_3_O_4_ nanoparticles

Fe_3_O_4_ nanoparticles were synthesized from ferric acetyl acetonate, 1-dodecanediol, oleic acid, oleylamine, diphenyl ether and *N*-(3-aminopropyl)^53^. A mixture of 2.023 g 1-dodecanediol (10 mM), 1.605 g oleylamine (6 mM) and 1.694 g oleic acid (6 mM) was added to 20 ml of 2 mM ferric acetyl acetonate in divinely ether. The solution was thoroughly degassed by purging with nitrogen. The solution was heated to 200 °C under vigorous stirring conditions for 30 min, and refluxed for another 30 min at 250 °C. The solution colour turned dark, indicating the formation of oleic-acid-capped Fe_3_O_4_ nanoparticles. The product was cooled to room temperature, followed by repeated washes in *n*-hexane and centrifugation to separate the nanoparticles, and finally dried in vacuum.

### Water-dispersible magnetic nanoparticles

A ligand exchange protocol^54^ was applied to obtain hydrophilic MAG nanoparticles with carboxylic functional groups (COO–) as capping agents. MAG nanoparticles were dispersed in *n*-hexane (∼20 mg ml^−1^) and added to a suspension of TAU in dichloromethane (20 mg ml^−1^). After vigorous stirring for 30 min, the particles were separated using a magnet.

### Synthesis of ferromagnetic NTs

Magnetic NTs were prepared in water according to the ISA method^52^: 40 mg of chrysotile powder was suspended in 8 ml of PBS (pH 7.4). An equally buffered solution of TAU-capped ferromagnetic nanoparticles (2 mg ml^−1^) was added slowly under stirring. Addition was stopped after 10 ml. At this point, the compound was repeatedly centrifuged and washed with water. Functionalized NTs were dried at 110 °C for 3 h. Fluorescent magnetic NTs (NT-MAG-Alexa 647) were prepared following the same procedure using Alexa Fluor 647 labelled NTs.

### Magnetic relaxivity measurements

NT-induced relaxation was performed on a 2 mg ml^−1^ NT solution in PBS performing up to five dilutions. A 0.5 T Bruker Minispec mq20 broadband spectrometer (1H Larmor frequency, 19.65 MHz) was used for magnetic relaxometry. Experiments were performed at 303 K (29.85 °C) ± 0.1 K using a BVT3000 Eurotherm nitrogen gas thermal apparatus. First, 120 μl of each solution was put in a tube (outside diameter, 10 mm) and positioned in the magnet. All samples were thermalized for 10 min before performing any experiment. The *T*_1_ relaxation time was measured using the saturation recovery pulse sequence, performing eight scans with a 0.1 s recycle delay and gain between 76 and 78 dB by acquiring 18 points with different recovery times. The relaxation time *T*_2_ was measured using the CPMG pulse sequence, acquiring eight scans with a 3 s recycle delay and a gain between 76 and 78 dB (refs. ^54–56^). Once all relaxation data were acquired, we plotted the inverse *R*_1_ = 1/*T*_1_ and *R*_2_ = 1/*T*_2_ as a function of the NT concentration.

### NT-MAG-Exo^myo^ release

Exo^myo^ were added overnight to a dispersion of NT-MAGs according to a maximal loading capacity of 5 × 10^9^ Exo^myo^ for 500 μg NT-MAG. The NT-MAG-Exo^myo^ suspension was centrifuged at 2,000*g* for 5 min and transferred into buffer solution at two different pH levels (6.5 and 7.4). Aliquots of NT-MAG-Exo^myo^ suspension were collected after 1, 6 and 24 h and centrifuged to precipitate NT-MAGs still carrying Exo^myo^. Supernatants containing the released CFSE exosomes were measured either with a Glomax microplate reader using excitation at 488 nm and an emission filter of 500–520 nm, or by NTA to evaluate the particle number.

### Static magnetic field tests

The static magnetic field used to drive the exosome nanocarriers was generated by a solid-state cylindrical neodymium magnet (N42, 0.5 T field) for the in vitro experiments and by a diameter (outer) 50 mm × diameter (inner) 30 mm × height 10 mm neodymium ring magnet (N42, 1.4 T field in the ring centre) for the in vivo experiments. Both magnets were purchased from Hangzhou Yang Yi Magnetics.

### In vivo bioluminescence

Two-dimensional optical imaging of bioluminescence was performed using a Bruker 2D U-OI system including a computer-controlled filter wheel with narrowband emission filters for multispectral bioluminescence, fluorescence and imaging. The intensity of the bioluminescence was represented using a colour scale. Images were acquired 24 h and 7 days after injection. Mice were imaged in a ventral position using epi-illumination 2D fluorescence imaging. An exposure time of 3 min and binning of 4 was used to maximize the sensitivity of the bioluminescence signal but avoid saturation of the detector. Images were acquired with two excitation filters, 472 nm for CFSE and 575 nm for Alexa 647, and emissions were collected at 520 nm and 615 nm, respectively. Images were generated using 2D Optical Living Image software.

### In vivo muscular MRI

A preclinical 7 T MRI scanner (Bruker, BioSpec 70/30 USR, Paravision 5.0) was equipped with 450/675 mT m^−1^ gradients (slow speed, 3,400–4,500 T m^−1^ s^−1^; rise time, 140 μs). The receiver was a rat heart phased-array coil with four internal preamplifiers, coupled with a 72 mm linear volume coil as the transmitter. The mice were induced under general anaesthesia using 1.5–2% isoflurane vaporized in 100% oxygen (flow, 1 l min^−1^). They were placed in a prone position with their hind limbs fixed at the centre of the coil. Respiration and body temperature were monitored and maintained at approximately 30 breaths per minute and 37 °C, respectively. Skeletal muscle was evaluated by the *T*_2_ relaxation time (*T*_2_-rt) as assessed by *T*_2_ mapping and fractional anisotropy using diffusion tensor imaging (DTI). Muscle *T*_2_ maps were obtained using a multislice–multiecho sequence with fat suppression (repetition time, 1,938 ms; 16 echo times, 10.73/171.68 ms; field of view, 20 mm × 20 mm; 256 × 256 matrix; spatial resolution, 0.078 × 0.078 mm per pixel; number of signal averages, 4) acquired in the axial plane (10 slices; thickness, 1 mm; gap, 0 mm). The DTI data were acquired using a SpinEcho-EPI (DTI-Epi) sequence with 30 diffusion gradient directions (repetition time, 3,750 ms; echo time, 33 ms; *b*-values for diffusion directions, 0–700 s mm^−^^2^; diffusion gradient duration, 4 ms; diffusion gradient separation, 20 ms; number of signal averages, 2). The DTI-Epi sequence shared the same geometric characteristics (field of view, 30 mm × 30 mm; 128 × 128 matrix; spatial resolution, 0.234 × 0.234 mm per pixel; 10 slices; slice thickness, 1 mm; gap, 0 mm).

### Endurance and muscle strength

Mice were placed on a transparent treadmill belt with a constant 10% slope and stepwise increasing rotation speed: 10 cm s^−1^ from 0 to 5 min, 15 cm s^−1^ from 5 to 10 min, 20 cm s^−1^ from 10 to 20 min, 25 cm s^−1^ from 20 to 30 min, and 30 cm s^−1^ from 30 to 35 min. Data were recorded at 20 cm s^−1^ (low intensity), 25 cm s^−1^ (intermediate intensity) and 30 cm s^−1^ (high intensity). The number of accumulated shocks was recorded at each time point. In the event of evident physical exhaustion before the end of the test, the animals were removed from the apparatus and an arbitrary value assigned based on the total distance travelled. Functional measurements were performed in a blind manner. The tetanic force of the TA muscle was determined as described previously^57^, normalized to the muscle CSA and expressed as kN m^−^^2^.

### Histology and immunofluorescence

TA muscle tissues were collected and frozen in liquid-nitrogen-cooled isopentane and cut on a cryostat into 10 µm slices. H&E staining was performed as described previously^57^. For immunofluorescence, slides were fixed with 4% paraformaldehyde for 10 min, permeabilized with 0.3% Triton X-100 for 15 min and incubated with 10% donkey serum or goat serum for 1 h to block non-specific binding. Next, the slides were incubated overnight at 4 °C with primary antibodies diluted in blocking solution. Slides were mounted using Prolong Gold Antifade Reagent with DAPI. A Leica TCS SP8 confocal or a Leica DMi8 fluorescence microscope were used to acquire images. The enzymatic activity of SDH was assayed by placing the slides in SDH incubating solution containing sodium succinate as a substrate and nitroblue tetrazolium for 1 h at 37 °C to visualize the reaction. Initially, slides were incubated for 10 s in 30–60–90–60–30% acetone solution, and then for 30 s in 80–90–100% ethanol solution for dehydration. Finally, they were incubated in 100% xylene for 1 min before mounting with DPX reagent on coverslips. For H&E, immunofluorescence and SDH staining, whole section images were captured by a Leica CTR6000 Laser microdissector. Hydroxyproline content was determined (in μg) per milligramme of muscle as a measure of fibrosis^58^. All chemicals are listed in Supplementary Tables 1 and 2.

### Image quantification and statistical analysis

Histological and immunofluorescence images were captured with a Leica CTR6000 microscope and a Leica TCS SP8 confocal microscope, respectively. Quantitative analyses were performed in ImageJ (NIH). The threshold colour plugin of ImageJ was used to quantify H&E and SDH staining as the percentage area over a fixed grid area. Data were analysed by GraphPad Prism and expressed as means ± s.d. To compare multiple groups means, we performed one-way analysis of variance (ANOVA) followed by Tukey’s multiple-comparison test or Kruskal–Wallis test followed by Dunn’s test.

### Single muscle fibre isolation

Single muscle fibres were isolated from TA muscles according to published protocols^59^. Briefly, the muscles were isolated from anaesthetized mice and incubated in in high-glucose DMEM containing 0.5% collagenase II for 3 h at 37 °C without shaking. After incubation, the muscles were transferred to prewarmed 60 mm dishes containing 5 ml DMEM supplemented with 10% horse serum. Residual connective tissue was removed with forceps, and the muscle was triturated using progressively smaller diameter glass pipettes to liberate single muscle fibres.

### RNA isolation and sequencing analysis

Macrophages and single muscle fibres were frozen immediately after sorting in liquid nitrogen and RNA extracted using an RNeasy Micro kit. RNAs were qualified and quantified on an Agilent Tapestation 2200 using a high-sensitivity RNA chip. To prepare the library, 150–300 ng of total RNA was reverse-transcribed using Illumina’s TruSeq stranded mRNA library preparation kit. Each sample was fitted with one of 96 adapters containing a different 8 base molecular barcode for high-level multiplexing.

Libraries were sequenced on an Illumina NovaSeq 6000. To ensure quality, the FASTQ files were checked with FastQC. We determined transcript/gene abundance using salmon v.1.10.2 and a specific transcriptome index (GRCm38). Normalization using edgeR v.3.42.0 was based on the read count matrix. Genes were considered expressed if their raw counts were >20 in at least two out of three replicates per condition. We used ggplot2 v.3.4.4 for volcano plot analysis of the RNA-seq expression data. Fold-changes between groups were calculated using the Bioconductor package EdgeR with the likelihood ratio test (http://www.bioconductor.org/packages/release/bioc/html/edgeR.html). Genes were considered differentially expressed if ∣log(fold change)∣ ≥ 1.5 (ref. ^60^). Differentially expressed genes were submitted to clusterProfiler v.4.8.2 for GO analysis^61^ in the GO Ontology database (v.2021-05-01). We used all genes expressed in the experiment and the GO terms ‘biological processes’, ‘cellular components’, ‘molecular functions’ as the annotation dataset. Significantly enriched GO terms were identified by an adjusted *P* < 0.05. GSEA was performed by dedicated software (release 4.2.3) in the Molecular Signatures Database (MsigDB). The ‘Hallmark’ annotated gene set collection was used for analysis of ranked gene lists. Data have been deposited in NCBI’s Gene Expression Omnibus and are accessible through GEO Series accession number GSE263457.

### Statistical analysis and reproducibility

No statistical methods were used to predetermine sample sizes, but our sample sizes are similar to those reported in previous publications^6,10^. The sample sizes and the specific statistical tests for each experiment are detailed in each figure caption. Statistical analysis was performed using Excel and GraphPad Prism. Statistical significance was set at *P* < 0.05.

## Online content

Any methods, additional references, Nature Portfolio reporting summaries, source data, extended data, supplementary information, acknowledgements, peer review information; details of author contributions and competing interests; and statements of data and code availability are available at 10.1038/s41565-024-01725-y.

## Supplementary information

Supplementary Information Supplementary Discussion 1–8, Methods, Figs. 1–10, Tables 1–3 and References.

Supplementary Data Source Data for Supplementary Fig. 1. Unprocessed western Blots and/or gels, statistical source data.

Supplementary Data Source Data for Supplementary Fig. 2. Unprocessed western Blots and/or gels, statistical source data.

Supplementary Data Source Data for Supplementary Fig. 4. Unprocessed western Blots and/or gels, statistical source data.

Supplementary Data Source Data for Supplementary Fig. 5. Unprocessed western Blots and/or gels, statistical source data.

Supplementary Data Source Data for Supplementary Fig. 6. Unprocessed western Blots and/or gels, statistical source data.

## Source data

Source Data Fig. 1 Unprocessed western Blots and/or gels, statistical source data.

Source Data Fig. 2 Unprocessed western Blots and/or gels, statistical source data.

Source Data Fig. 4 Statistical source data.

Source Data Fig. 5 Unprocessed western Blots and/or gels, statistical source data.

Source Data Extended Data Fig. 1 Statistical source data.

## Data Availability

The MS data have been deposited to the ProteomeXchange Consortium via the PRIDE partner repository with the dataset identifier ftp://MSV000094590@massive.ucsd.edu. Transcriptomic data have been deposited in NCBI’s Gene Expression Omnibus and are accessible via GEO Series accession number GSE263457. Source data are provided with this paper.
