# Supplementary material for: Magnetic-field-driven targeting of exosomes modulates immune and metabolic changes in dystrophic muscle
Source: Nat Nanotechnol. 2024 Jul 22;19(10):1532–43. doi: 10.1038/s41565-024-01725-y (PMC11486659; doi:10.1038/s41565-024-01725-y)
Supplement: Supplementary file 1 — Supplementary Discussion 1–8, Methods, Figs. 1–10, Tables 1–3 and References. [file 41565_2024_1725_MOESM1_ESM.pdf]

nature nanotechnology

Supplementary information

https://doi.org/10.1038/s41565-024-01725-y

Magnetic-field-driven targeting of exosomes modulates immune and metabolic changes in dystrophic muscle

In the format provided by the authors and unedited

Table of Contents

Supplementary Discussions

Supplementary Methods

Supplementary Figures

Supplementary Tables

References

Supplementary Discussions

Supplementary Discussion 1

We freshly isolated Ly6C+ macrophages through fluorescence-activated cell sorting (FACS) from infiltrated muscles of 3-month-old mdx mice and cultured them in vitro for 5 days in the presence of 10^8 Exo^{myo} or 10^8 Exo^{C2C12}. The untreated macrophages demonstrated elevated expression of canonical inflammatory M1 genes (*iNOS, Cxcl 10, CD 86, TNF*α). In contrast, macrophages treated with Exo^{myo} exhibited heightened expression of anti-inflammatory M2-associated genes (*Ym1, Ccl 22*) and a concurrent downregulation of M1 genes when compared to macrophages treated with 10^8 Exo^{C2C12} (Fig. 1h).

Supplementary Discussion 2

Supernatants were collected from dissociated TA muscles and used for magnetic capture of CD63-positive exosomes. The retention of Exo^{myo} in dystrophic muscle was confirmed by co-detection of exosome markers CD9, CD81, and CFSE by FACS. Twenty-four hours after administration, intramuscular injection resulted in a higher percentage of CFSE+ exosomes (Exo^{myo}: 47.1%; Exo^{C2C12}: 34.4% %) than intravenously delivery (Exo^{myo}: 1.68 %; Exo^{C2C12}: 1.01%) Slow clearances of intramuscularly injected CFSE+ exosomes were observed on day 7 (Exo^{Myo}: 9.1%; Exo^{C2C12}: 3.8%; versus intravenously delivery, Exo^{Myo}: 0.47%; Exo^{C2C12}: 0.19%) (Fig. 2a).

Supplementary Discussion 3

The circuit was designed with a bifurcation in two branches connected to a peristaltic pump, allowing for a consistent flow rate of 15 to 80 ml/min (Extended Data Figure 1e). One of the branches was specifically linked to an external reservoir through a septum covered by murine aortic wall. To drive the migration of NT-MAG-Exo^{myo} across the wall, an external static 0.5 T magnetic field was applied to the connected branch-vessel-reservoir (Extended Data Figure 1e). We tested NT-MAG-Alexa 647 and the NT-MAG-Exo^{myo} system with a 25 ml/min flow rate. The blood that migrated through the aortic septum into the reservoir and the remaining blood within the circuit were collected 30 min and 1 h after magnetic field exposure; $77.5 \pm 5.1\%$ of circulating NT-MAG-Alexa 647 successfully migrated through the aortic wall (Extended Data Figure 1f).

Supplementary Discussion 4

The number of shocks was greatly reduced in NT-MAG- Exo^{myo} III mdx mice compared to the other groups, starting 10 days after treatment, for intermediate and high running speeds, with improvement maintained 20 and 30 days after the first injection. From 30 days, NT-MAG- Exo^{myo} III mdx mice had a comparable treadmill

performance as C57Bl mice, at any speed, suggesting that weekly magnetic delivery of NT-MAG- Exo^{myo} could help improve the fatigue resistance of mdx mice (Supplementary Figure 4 a-c)

Supplementary Discussion 5

NT-MAG-Exo^{myo} alleviated myopathy of mdx marked by increased CSA in injected TA muscle. Muscle CSA increased accordingly with NT-MAG-Exo^{myo} number of doses, with NT-MAG-Exo^{myo} III displaying the higher area. Values of frequency distribution confirmed the smaller area of myofibers in mdx mice that received vehicle and NT-MAG-Exo^{myo} I administration (Veh: median = 1067.92; 25% percentile = 558.40; 75% percentile = 2250.04; NT-MAG-Exo^{myo} I: median = 1533.91; 25% percentile = 863.48; 75% percentile = 2277.29; NT-MAG-Exo^{myo} II: median = 1643.96; 25% percentile = 818.44; 75% percentile = 2540.32; NT-MAG-Exo^{myo} III: median = 1947.21; 25% percentile = 1260. 39; 75% percentile = 2681.62) (Supplementary Fig. 5a).

Supplementary Discussion 6

The number of shocks was significantly reduced in NT-MAG-Exo^{myo} III mdx mice compared to NT-Exo^{myo} III mdx mice, starting from 10 days after treatment, for intermediate and high running speeds. The improvement was maintained 20 and 30 days after the first injection (Fig. 5a).

Supplementary Discussion 7

Among 1279 differentially regulated genes, 488 were up-regulated in iMACs from NT-MAG-Exo^{myo} III, 259 from NT-MAG-Exo^{myo} II, and 85 from NT-MAG-Exo^{myo} I (fold-change ≥1.5, P≤0.05). Conversely, iMACs from NT-MAG-Exo^{myo} I had 176 down-regulated genes, of which 110 and 102 were shared with NT-MAG-Exo^{myo} II and NT-MAG-Exo^{myo} III, respectively (fold-change ≥1.5, P≤0.05) (Extended Data Fig. 2a-c). iMACs from NT-MAG-Exo^{myo} III had 4046 differentially regulated genes compared to the NT-Exo^{Myo} group. The iMAC GO pathway signatures were highlighted in the NT-MAG-Exo^{myo} III group, with significant up-regulation of "generation of metabolites precursors and energies" (GO:0006091) "energy derivation by oxidation" (GO:0015980), "aerobic respiration" (GO:0009060), and "OXPHOS" (GO:0006119), whereas "chemotaxis" (GO:0006935), "T cell activation" (GO:0042110), and "leukocyte migration" (GO:0050900) were down-regulated (Extended Data Fig. 2d). Down-regulated GO pathways included biological processes related to "blood vessel morphogenesis" (GO:0048514) and "angiogenesis' (GO:0001525) in the NT-MAG-Exo^{myo} I group, "tissue morphogenesis" (GO:0048729) and "cell differentiation" (GO: 0030154) in the NT-MAG-Exo^{myo} II group, and "antigen processing and presentation via MHC" for the response to molecules of bacterial origin (GO:0002237) and to an external stimulus (GO:0032103) in the NT-MAG-Exo^{myo} III group

(Extended Data Fig. 2d). The mitochondrial/oxidative pathway was up-regulated in iMACs after NT-MAG-Exo^{myo} treatment in all groups. The GO pathways up-regulated in iMACs in the NT-Exo^{myo} III group were "purine nucleotide metabolic process" and "ribonucleoside triphosphate metabolic process", whereas "regulation of translation", "regulation of mRNA metabolic process," and negative regulation of translation" were down-regulated (Supplementary Fig. 9a). GSEA and Volcano analysis of NT-MAG-Exo^{Myo} III group showed upregulation of oxidative genes (*Akt2, Pdpk1, Birc3, Snca, Gadd45gip1, Cox7a1, Sdhc, Ndufs4, Ndufa12, Uqcrb, Coq7, Ndufb3, Coq9*) (Supplementary Fig. 7 and Supplementary Fig. 8a). De-regulated KEGG pathways in iMACs from NT-MAG-Exo^{myo} I mice were related to inflammatory signaling ("Malaria", "African trypanosomiasis") but also included genes in the GO "motor/cardiac muscle contraction" pathways (*Actc1, Myl3, Tpm2, Tpm1, Hrc*) (Supplementary Fig 7). Up-regulated KEGG pathways in iMACs from the NT-MAG-Exo^{myo} II and NT-MAG-Exo^{myo} III groups were also related to survival/apoptosis and oxidative metabolism ("Parkinson disease," "Alzheimer disease," "Prion disease," "Diabetic cardiomyopathy," and "Oxidative phosphorylation") (Supplementary Fig. 7). Down-regulated KEGG pathways demonstrated reduced macrophage chemotaxis and phagocytic function in iMACs from the NT-MAG-Exo^{myo} I group ("cGMP−PKG signaling pathway" and "Phagosome"), whereas NT-MAG-Exo^{myo} II and NT-MAG-Exo^{myo} III iMACs had down-regulated KEGG pathways related to macrophage receptors sensing products of adaptive immune cells ("Cytokine-cytokine receptor interaction," Cell adhesion molecules," "Coronavirus disease," "Antigen processing and presentation," "Malaria," and "Allograft rejection") (Supplementary Fig. 7). GSEA and Volcano analysis of iMAC from the comparison of NT-MAG-Exo^{myo} III vs. NT-Exo^{myo} III confirmed up-regulation of genes involved in reprogramming metabolism and mRNA translation of M2 macrophages (*Rbp1, Maff, Shank1, Has1, Cebpd, Fosb*) in NT-MAG-Exo^{myo} III.

Supplementary Discussion 8

Most differentially up-regulated genes from single muscle fibers isolated from NT-MAG-Exo^{myo} II and NT-MAG-Exo^{myo} III mice were classified into "aerobic respiration," "cellular respiration," and "respiratory electron transport" (fold-change $\geq$1.5, P$\leq$0.05) (Extended Data Fig. 3d). GO pathway analysis also revealed up-regulation of "humoral immune response" (GO:0006959) and "extracellular matrix organization" (GO:0030198) (fold-change $\geq$1.5, P$\leq$0.05) in single muscle fibers from NT-MAG-CFSE-Exo^{myo} II and NT-MAG-CFSE-Exo^{myo} III mice (Extended Data Fig. 3d). Conversely, differentially down-regulated genes in the single muscle fiber groups were associated with "blood vessel morphogenesis" (GO:0048514), "angiogenesis" (GO:0001525), "striated muscle cell differentiation" (GO:0051146), and "muscle tissue development" (GO:0048514) (fold-change $\geq$1.5, P$\leq$0.05) (Extended Data Fig. 3d). Genes analysis of myofibers from NT-MAG-Exo^{Myo} II and NT-MAG-Exo^{Myo} III group revealed an enrichment for skeletal muscle contraction and transition between fast and slow fiber genes, such as *Cox7a1, TPM2, Myl1, Myh11,* and *Tnnc2* involved in troponin and myosin complex, myofibril actin filament and muscle thin filament tropomyosin (Supplementary Fig. 10). In addition, we detected a downregulation in the expression profile of *S100a8, S100a9, Col2a1*and

several other genes involved in extracellular matrix response to inflammatory stimuli in myofibers from NT-MAG-Exo^{Myo} III compared to NT-Exo^{Myo} III group (Supplementary Fig. 10).

Supplementary Methods

Fluorescence-activated cell sorting of muscle-derived cells

Hind limb muscles (vastus medialis, vastus lateralis, rectus femoris, biceps femoris, adductors, gastrocnemius, and TA) from mdx mice were cut into small pieces and digested in an enzyme solution [dispase (11 U/ml) and collagenase II (2000 U/ml)] for a total of 1 hour and 40 minutes at 37°C with gentle shaking. The digested tissue was then passed through 19-gauge needles and filtered through a 70-µm nylon cell strainer to obtain a single-cell suspension. For muscle SC isolation, the washed pellet was resuspended in 500 µl PBS and transferred to 5 ml FACS tubes. The following primary antibodies were added to the tubes [1]: CD45 FITC, CD31 FITC, F4/80 FITC; anti-alpha7 integrin PE-Vio770 (ITGA 7), Sca1 PE, CD 34 APC. Viability was evaluated by 7AAD staining. Samples were gently vortexed and incubated on ice for 15 min. BSA (0.1% in PBS) was added to each tube after staining and cells pelleted at 500×g for 5 min at 4°C. Single color tubes were run as controls. FACS was achieved with FACSAria Fusion (BD Biosciences). For macrophage sorting, hind limb muscles from mdx mice were collected, cut into 1-mm2 pieces, and digested for 1 h at 37°C in 0.2 mg/ml Liberase suspended in RPMI with light shaking. The digested tissue was diluted in 10 ml freshly made F10 with 20% FBS and flushed through an 18G needle to dissociate undigested tissues. After filtration through 70-µm mesh filters and centrifugation at 500 g for 10 min (Eppendorf, 5810R), the cell pellet was washed in PBS 1X and centrifuged again at 500 g for 10 min. Mononuclear cells were isolated by density gradient centrifugation using 40% and 80% Optiprep. Circulating macrophages were isolated by FACS using the following antibodies: BV510-conjugated anti-mouse CD45; PE-conjugated anti-mouse CD11b antibody, APC-conjugated anti-mouse F4/80, and PE Cy7-conjugated Ly6C. Macrophages were gated according to their sizes and granularity defined in the forward light scatter and side light scatter plot. iMACs were sorted based on their F4/80/CD11b profiles in the CD45+ population and Ly6C positivity. For RNA-seq analysis, Ly6C+ macrophages were gated and sorted for CFSE staining. All chemicals and antibodies are listed in Supplementary Table 1 and Supplementary Table 2.

Real-time quantitative polymerase chain reaction

Total RNA was extracted using the RNAprep Pure Cell Kit and reverse-transcribed using PrimeScript RT Master Mix. The FastStart Universal SYBR Green Master was added to each reaction, along with cDNA and specific primers to a total volume of 10 µl. Real-time quantitative PCR was performed on an ABI Prism 7900HT. The primer sequences are available in Supplementary Table 3.

Western blotting

Total proteins were isolated from the TA muscle as described previously [2]. Samples were resolved on stain-free 4-15% polyacrylamide gels and transferred to nitrocellulose membranes. Filters were incubated overnight with antibodies and bands were normalized for total protein loading (visualized by stain free technology, in the Chemidoc system, Biorad) (https://www.bio-rad.com/it-it/product/image-lab-software).

Ex vivo magnetic field delivery

A two-way blood circulation simulator was designed and printed with white PLA filament using the Creative printer model Ender plus 5. By combining a mold and a counter mold, we moved on to the phase of pouring two-component transparent silicone with Shore 40 coating (Hoffmann Mineral). After 1 day of polymerization, the prototype was dipped in dichloromethane to melt the PLA mold and countermold. After complete dissolution of the polymer, drying, and cleaning, the desired system was obtained. To simulate blood viscosity, we used a mixture of PBS (64.86%), glycerol (35.12%), and xanthan (0.02%) [3]. We built a simulator with an inlet and outlet with a diameter of 4 mm and two circulation routes of 2 mm, the latter with an appropriate length and distance from the static magnet employed. We injected 2 mg of NT-MAG stained with Alexa 647 or Exo^{Myo} into the circulation system (25 ml of total blood). Aortas were harvested from 3-month-old C57Bl mice [4] and cut into 2 mm × 20 mm pieces. The aortic tissue was housed in a reservoir to collect extravasating liquid from the circuit. After 30 min, artificial blood passing through the aorta was collected and the Alexa 647 fluorescence measured with Glomax to obtain the milligrams of NTs able to cross the barrier. The same experiment was performed in the presence and absence of an external magnet. A straight calibration line was made to calculate the amount of the sample starting from the Glomax value. Confocal imaging of the aorta was performed to detect NT-MAG-647 and NT-MAG-Exo^{myo} entrapped within tissues. For laminin staining, the aorta was fixed in 4% PFA for 10 min and quenched with 0.1M glycine for 10 min. The tissue was permeabilized with 0.5% Triton-X 100 for 5 min and nonspecific staining blocked with 10% normal goat serum for 2 h at room temperature. The aortic tissue was incubated overnight at 4°C with rabbit anti-mouse laminin antibody diluted 1:50 in blocking solution. Donkey anti-rabbit secondary antibody was diluted 1:200 in PBS 1X and added for 1 h at room temperature. Vector True view was used to remove autofluorescence as directed. Stained aortic tissue was transferred into a chamber slide well (MatTek) with PBS 1X. Representative Z-stack images were captured by a Leica SP 8 confocal microscope equipped with a white laser.

Supplementary Figures and Legends

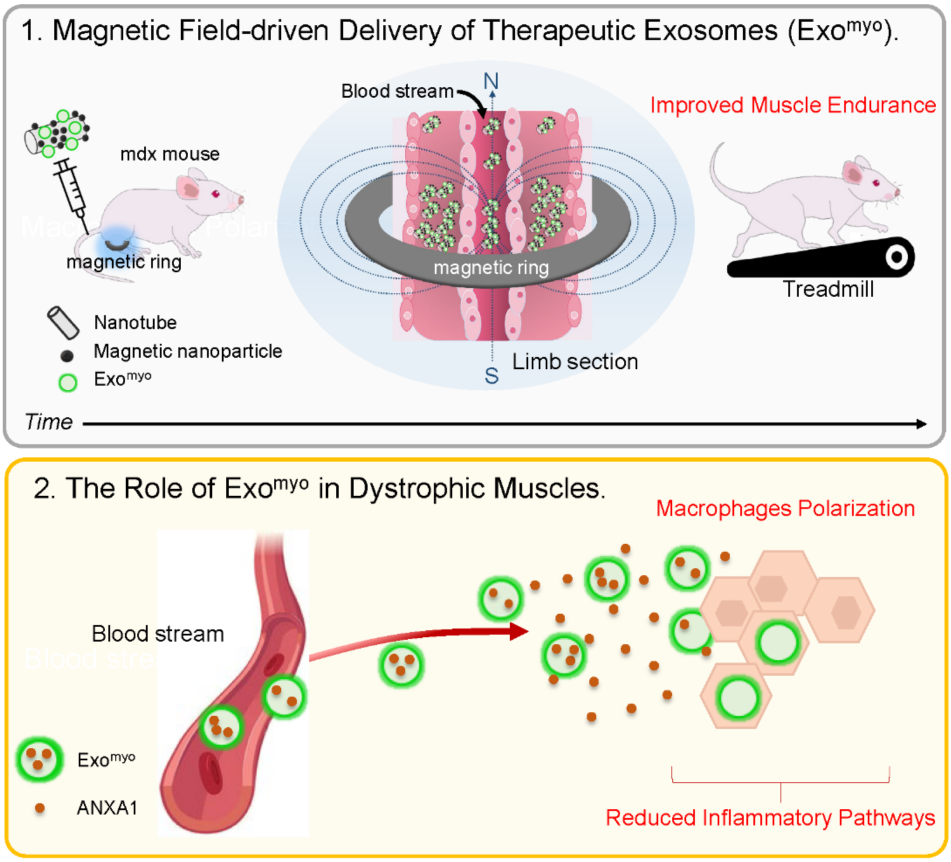

Figure sketch of the proposed strategy for the controlled delivery of therapeutic exosomes. Magnetic field–sensitive nanocarriers composed by biocompatible nanotubes decorated with ferromagnetic nanoparticles, transport the exosomes through the blood vessel, enabling their localization into skeletal muscles by an applied external magnetic and thereby contributing to the amelioration of Duchenne muscular dystrophy (DMD) myopathy.

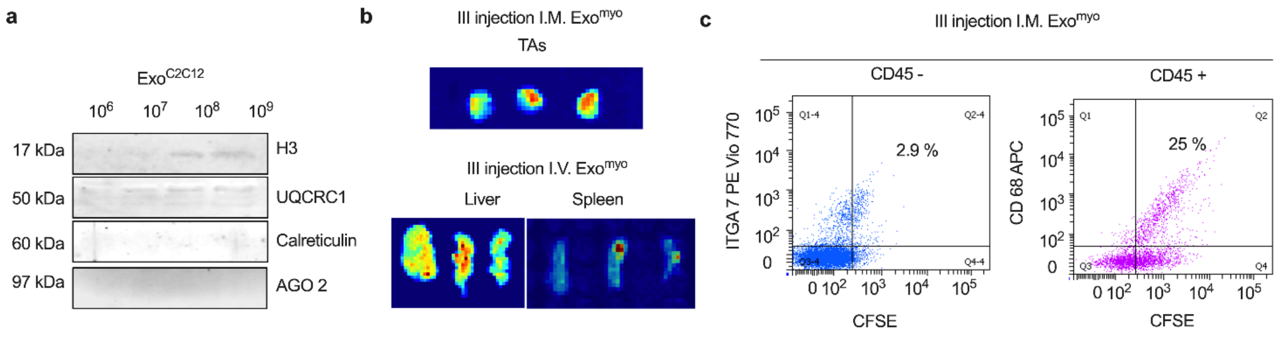

Supplementary Figure 1. Repeated intramuscular injections increases bioavailability of Exo^{myo} in dystrophic muscle tissues. a,

a, Cropped Western blot image of staining with nuclear (H3), mitochondrial (UQCRC1), endoplasmic reticulum (Calreticulin), and microvesiscles (AGO2) in 10^6, 10^7, 10^8 and 10^9 Exo^{C2C12} samples. Representative images from *n*=3 independent experiments are shown. **b**, Optical bioluminescent imaging of CFSE signal in the TAs of 3 months old mdx mice that received three Exo^{myo} doses (upper panel). CFSE accumulation in spleen and liver of mice treated with three I.V. doses of Exo^{myo} (bottom panel). *n* = 6 mice per group **c**, Representative cytofluorimetric analysis of supernatants collected from dissociated hind limb muscle from mdx mice injected with three I.M. doses of Exo^{myo}. *n*=3 samples per group.

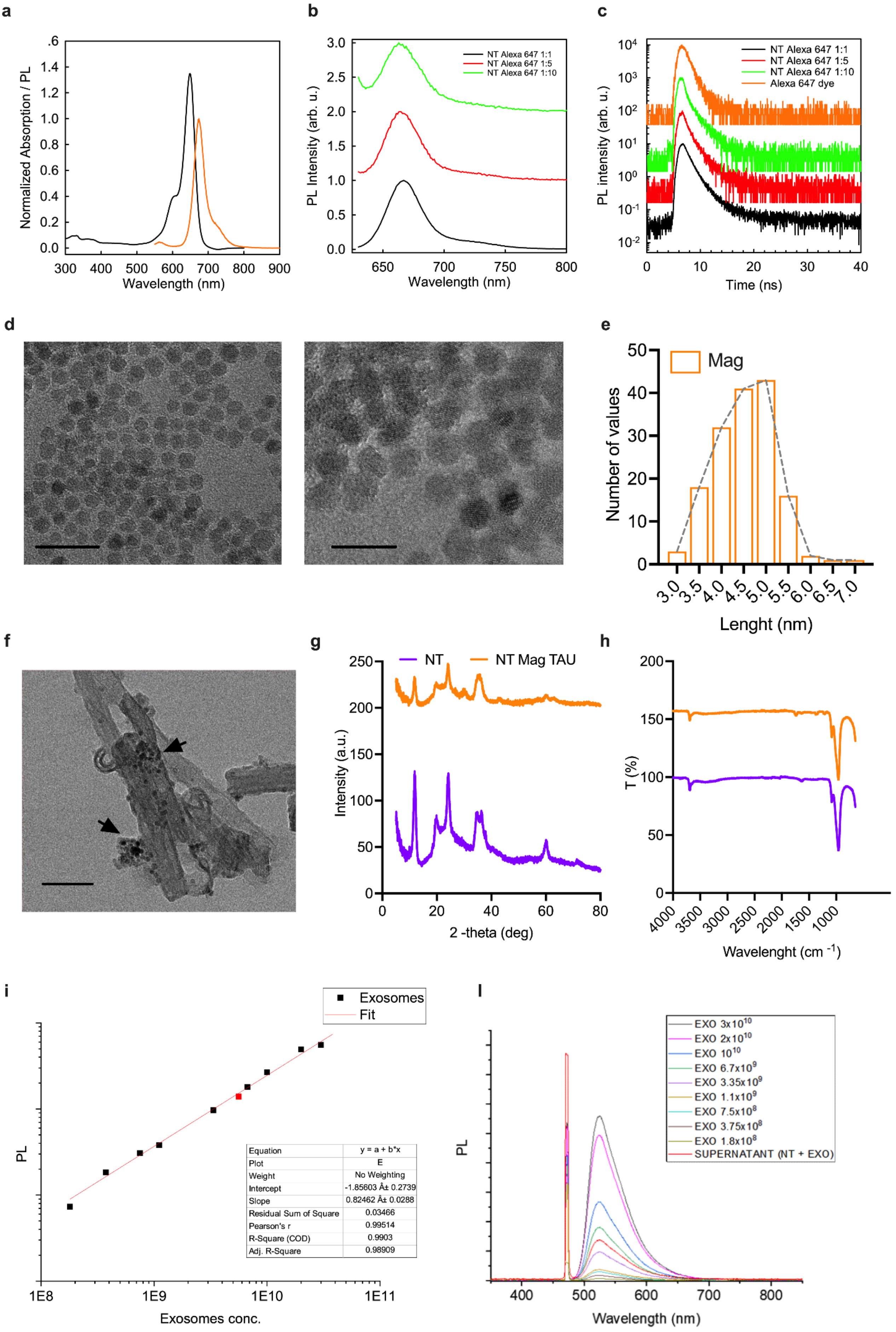
a
Normalized Absorption / PL
Wavelength (nm)
b
NT Alexa 647 1:1
NT Alexa 647 1:5
NT Alexa 647 1:10
PL Intensity (arb. u.)
Wavelength (nm)
c
NT Alexa 647 1:1
NT Alexa 647 1:5
NT Alexa 647 1:10
Alexa 647 dye
PL intensity (arb. u.)
Time (ns)
d
e
Mag
Number of values
Lenght (nm)
f
g
NT
NT Mag TAU
Intensity (a.u.)
2 -theta (deg)
h
T (%)
Wavelenght (cm -1)
i
Exosomes
Fit
PL
Exosomes conc.
Equation y = a + b*x
Plot E
Weight No Weighting
Intercept -1.85603 Â± 0.2739
Slope 0.82462 Â± 0.0288
Residual Sum of Square 0.03466
Pearson's r 0.99514
R-Square (COD) 0.9903
Adj. R-Square 0.98909
l
EXO 3x10^10
EXO 2x10^10
EXO 10^10
EXO 6.7x10^9
EXO 3.35x10^9
EXO 1.1x10^9
EXO 7.5x10^8
EXO 3.75x10^8
EXO 1.8x10^8
SUPERNATANT (NT + EXO)
PL
Wavelength (nm)

Supplementary Figure 2. Decoration of nanocarriers: **a**, Absorption, and photoluminescence (PL) spectra Alexafluor 647 in PBS under excitation at 405 nm. **b**, PL spectra of NTs stained with Alexafluor 647 at different coverage level in PBS dispersion under excitation at 405 nm. **c**, Time-resolved PL spectra are recorded at the maximum of the PL spectrum under pulsed excitation at 405 nm. No changes in the dye PL spectra and lifetime were observed by comparing the single molecule emission with the stained dye emission, indicating the Alexafluor 647 properties are preserved upon decoration of NT surfaces. **Characterization of decorated nanocarriers: d**, Transmission electronic microscope (TEM) image of ferromagnetic nanoparticles capped by tetramethyl ammonium 11-aminoundecanoate (scale bar 30 nm on the left and 10 nm on the right). **e**, Size distribution of the ferromagnetic nanoparticle diameter obtained by the analysis of TEM images. **Characterization of ferromagnetic nanoparticles**: **f,** Transmission electronic microscope (TEM) image of NTs functionalized with ferromagnetic nanoparticles capped by tetramethyl ammonium 11-aminoundecanoate (scale bar 100 nm). **g**, Powder X-ray diffraction patterns of bare NTs and NTs functionalized with ferromagnetic nanoparticles (NT-MAG-TAU). **h,** Attenuated reflectance FT-IR spectra of NTs and NT-MAG-TAU. **Exo^{myo} decoration of ferromagnetic nanocarriers**: we recorded a calibration curve (**i**) by studying the intensity of the PL emission from CFSE-Exo^{myo} as function of the exosomes concentration in PBS upon excitation at 473 nm (**l**). The amounts of exosomes that are effectively attached on NTs surfaces can be therefore calculated by measuring the intensity of the fluorescence recorded from the supernatant, from a NTs dispersion where NTS-MAG-Exo^{myo} has been sorted by using a static magnet. The supernatant fluorescence comes for the free exosomes that remain in solution. The maximum loading of exosomes has been archived by mixing 500µg di of NTs-MAG with 10^{10} exosomes in PBS. After 24h the NTs has been separated. The red dot in panel a is the intensity of the supernatant fluorescence ascribed to free exosomes in dispersion exosomes. By difference we therefore calculated the number of attached exosomes as high as 5×10^{9}.

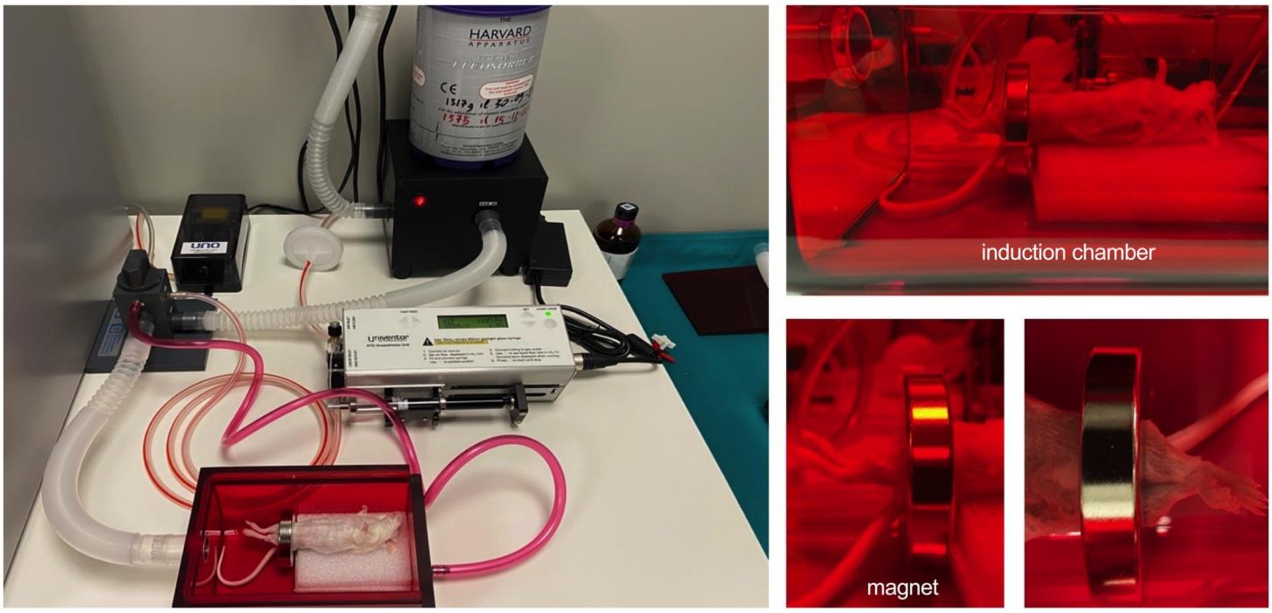

Supplementary Figure 3. Induction Chamber for Magnetic delivery of NT-MAG-Exo^{myo}. Representative digital image of the induction chamber (in red) and the gas scavenging unit used to administer inhalation anesthesia to murine models. The induction chamber was made of 10 mm thick acrylic and has an inlet port for the gas mixture on one side and a ø23mm outlet for the exhaust diffuser on the opposite side. The chamber's lid can be vertically slid open to access the interior. Once the animal is anesthetized, the lid of the induction box is slightly opened, and a magnet is placed around the hind limbs of the supine-positioned mouse (inset images). The caudal vein injection procedure is performed with the magnet in place for 30 minutes, and the induction chamber lid is partially opened only during the injection procedure.

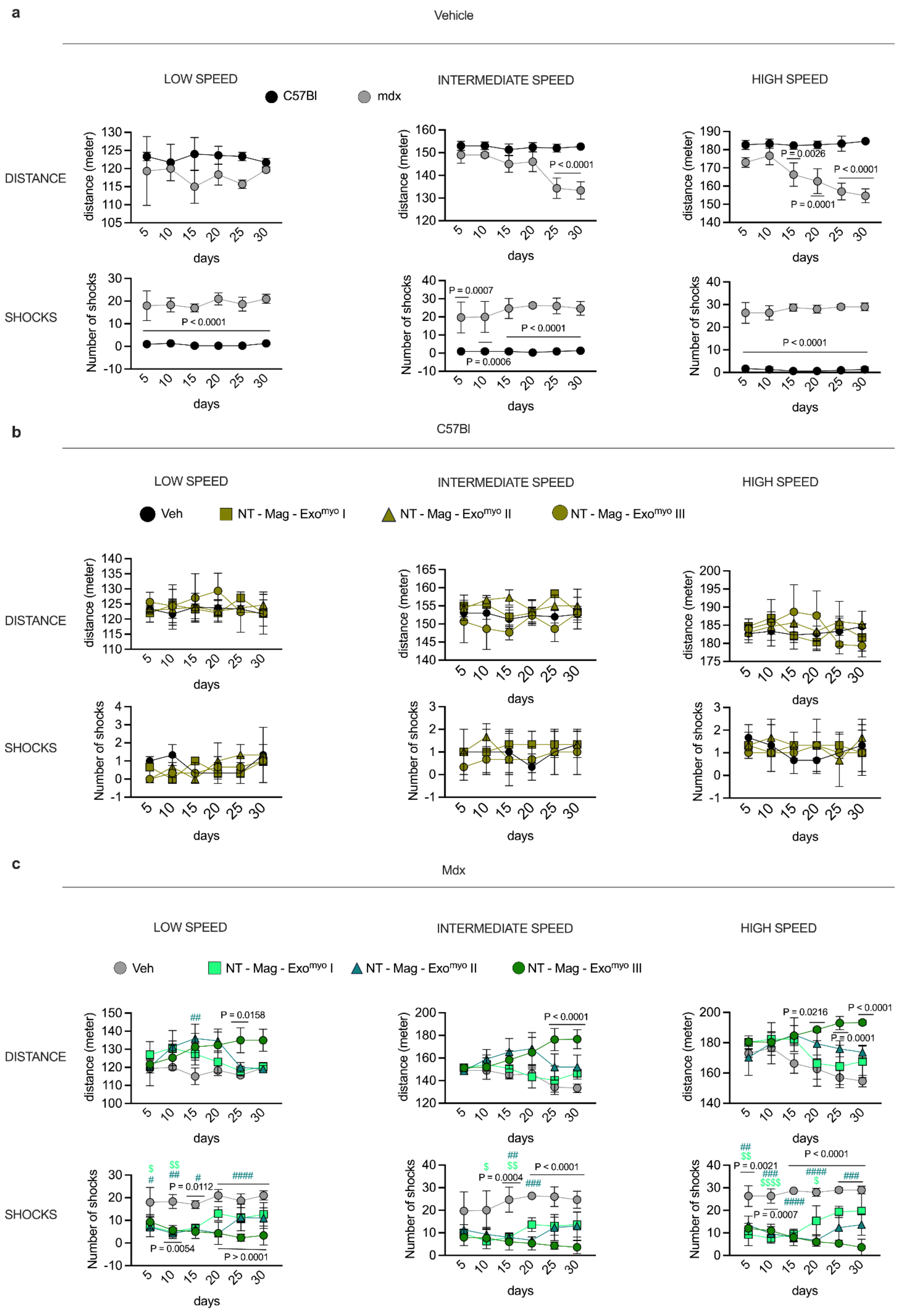
a
Vehicle
LOW SPEED
INTERMEDIATE SPEED
HIGH SPEED
C57Bl
mdx
DISTANCE
SHOCKS
distance (meter)
Number of shocks
days
P < 0.0001
P = 0.0026
P = 0.0001
P = 0.0007
P = 0.0006
b
C57Bl
Veh
NT - Mag - Exo^{myo} I
NT - Mag - Exo^{myo} II
NT - Mag - Exo^{myo} III
c
Mdx
P = 0.0158
P = 0.0112
P = 0.0054
P > 0.0001
P = 0.0004
P = 0.0216
P = 0.0021
P = 0.0007
##
$$
#
####
###
$$$$

Supplementary Figure 4. Functional measures of Exo^{myo}-treated mice.

a, Treadmill performance of (**a**) vehicle administered 3 months old C57Bl and mdx mice. **b – c,** Treadmill performance of C57Bl (**b**) and mdx (**c**) mice systemically injected with different doses of NT-MAG-Exo^{myo} delivered once a week through magnet positioning around limb muscles: NT-MAG-Exo^{myo} I, receiving one injection; NT-MAG-Exo^{myo} II, receiving two injections; NT-MAG-Exo^{myo} III, receiving three injections. All mice were sacrificed after 30 days from the first injection. One group of mdx mice received three I.V. injection of vehicle (PBS), once a week. Data were recorded at 20 cm/sec (low intensity), 25 cm/sec (intermediate intensity), and 30 cm/sec (high intensity). Results from the first injection to the sacrifice day are expressed as average distance and number of shocks ± SD; two-way ANOVA followed by the post hoc Tukey multiple comparison test. $n = 3$ mice per group. For comparison between NT-MAG-Exo^{myo} I ($) or NT-MAG-Exo^{myo} II (#) treated mice to vehicle treated mdx mice: $ or # P < 0.05, $$ or ## P < 0.01, $$$ or ### P < 0.001 and $$$$ or #### P < 0.0001; exact P values are reported for vehicle administered C57Bl mice compared to vehicle administered mdx mice, and NT-MAG-Exo^{myo} III administered mdx mice compared to vehicle condition.

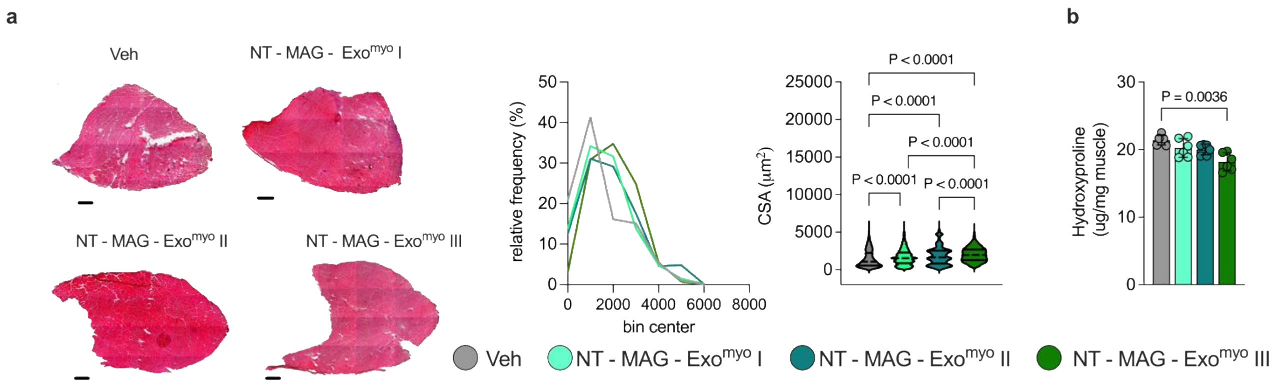

Supplementary Figure 5. Repeated systemic injections of NT-MAG-Exo^{myo} ameliorate the myofiber cross-sectional area (CSA) and reduce fibrosis in dystrophic muscle tissues.

a, Representative images of TAs stained with HE and quantification of myofiber cross sectional area and relative frequency. Scale bars for HE: 200 μm. For morphometric analysis, images were quantified using Image J software. For each group, H&E stained images from n = 4 mice were counted: n = 3640 myofiber number for PBS (Veh) treated mice, n = 3292 for NT-MAG-Exo^{myo} I, and n = 3304 for NT-MAG-Exo^{myo} II, and n = 3294 for NT-MAG-Exo^{myo} III injected mice. Violin plots showing CSA median (dotted lines) and quartiles (solid lines); Kruskal Wallis with Dunn's multiple-comparison test. **b**, Fibrosis was quantified using hydroxyproline assay from TAs isolated from mice injected with different doses of NT-MAG-Exo^{myo}; n = 3 samples per group, in n = 2 independent experiments. Statistical analysis was performed using one-way ANOVA followed by post hoc Tukey multiple comparison test.

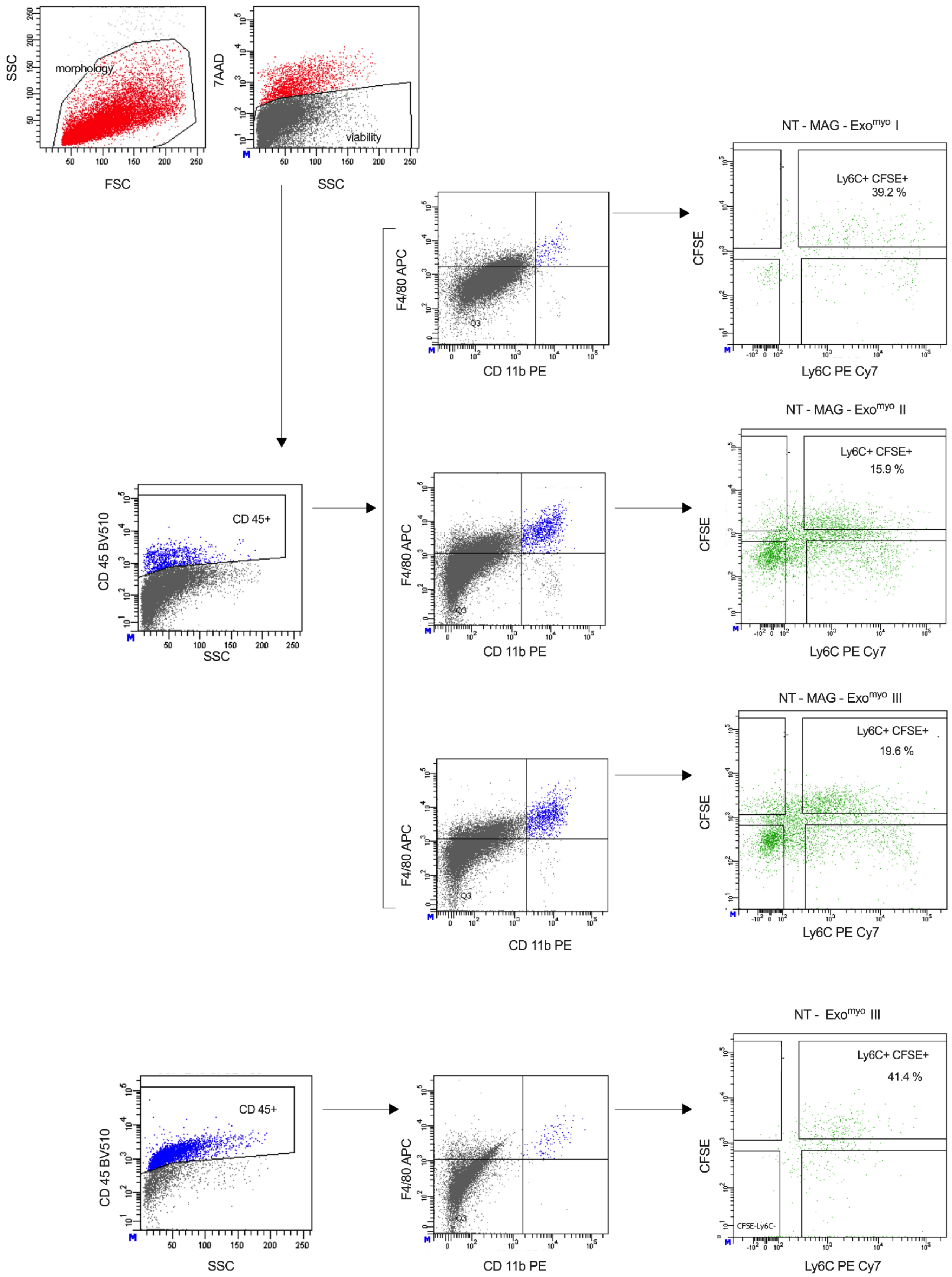

SSC
morphology
FSC
7AAD
viability
SSC
CD 45 BV510
CD 45+
SSC
F4/80 APC
CD 11b PE
Q3
NT - MAG - Exo^myo I
Ly6C+ CFSE+
39.2 %
NT - MAG - Exo^myo II
Ly6C+ CFSE+
15.9 %
NT - MAG - Exo^myo III
Ly6C+ CFSE+
19.6 %
CFSE
Ly6C PE Cy7
CD 45 BV510
CD 45+
SSC
F4/80 APC
CD 11b PE
NT - Exo^myo III
Ly6C+ CFSE+
41.4 %
CFSE-Ly6C-
CFSE
Ly6C PE Cy7

Supplementary Figure 6. Repeated systemic injections of NT-MAG-Exo^{myo} decrease the number of Ly6C+ infiltrating macrophages in dystrophic muscle tissues.

Muscle infiltrating macrophages were isolated by FACS Aria II as Ly6C+CFSE+ cells (iMAC) from 3 months old mdx mice systemically injected with different doses of NT-MAG-Exo^{myo} (NT-MAG-Exo^{myo} I, receiving one injection; NT-MAG-Exo^{myo} II, receiving two injections; NT-MAG-Exo^{myo} III, receiving three injections), and with three doses of non-magnetic NT-Exo^{myo} (NT-Exo^{myo} III) delivered through magnet positioning around murine muscles. All mice were sacrificed after 30 days from the first injection. Mononuclear cells from muscle were isolated through Optiprep gradient and labeled with BV510 conjugated CD 45, APC conjugated F4/80, PE conjugates CD 11b, and PE Cy7 conjugated Ly6C antibodies. Macrophages were gated according to their sizes and granularity defined in the forward light scatter (FSC) and side light scatter (SSC) plot. iMACs were sorted based on their F4/80/CD 11b profiles in the CD45+ population. Within this cell population, iMAC were then finally sorted for Ly6C and CFSE positivity. Representative gating strategies for n = 3 mice per group.

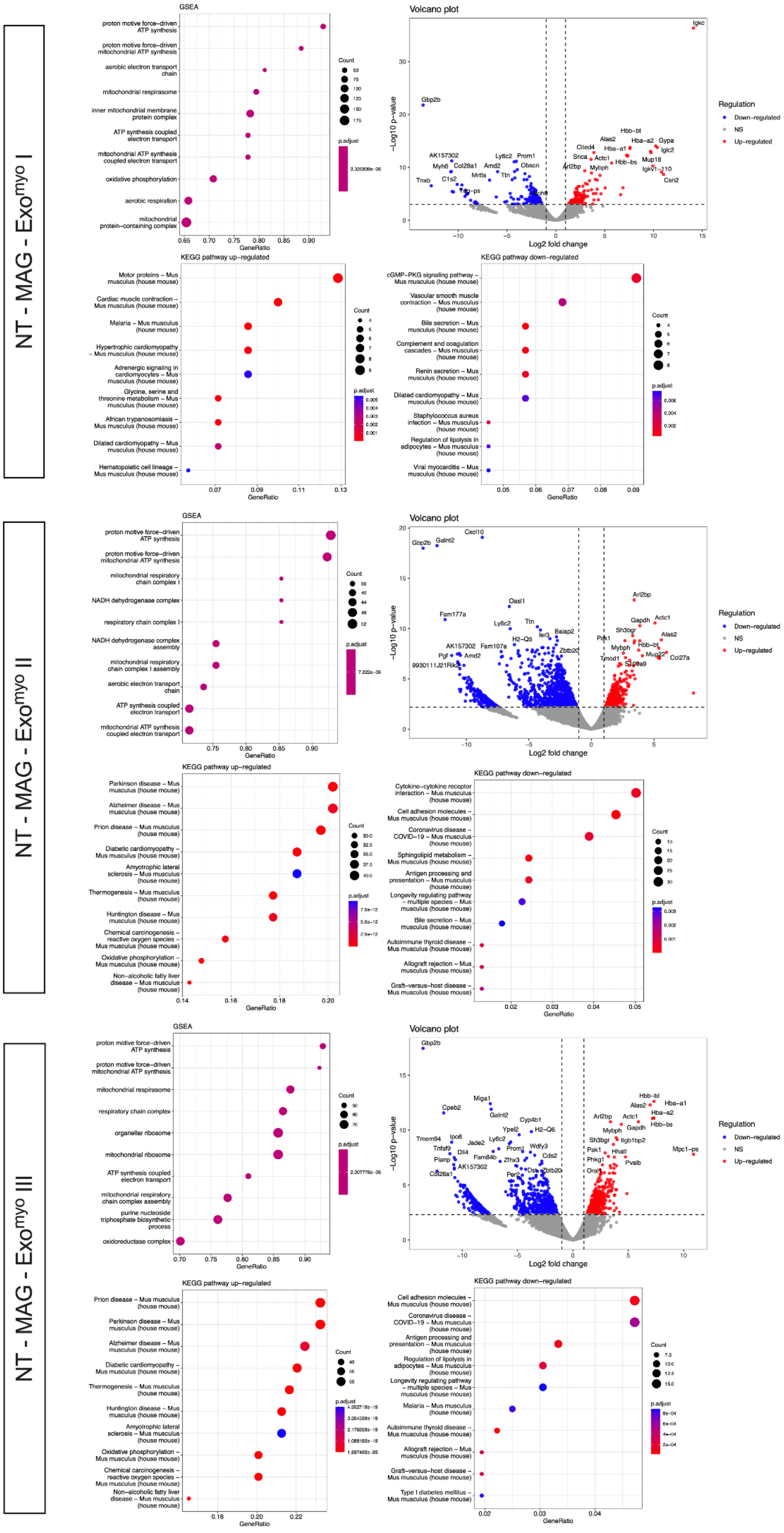
NT - MAG - Exo^{myo} I
NT - MAG - Exo^{myo} II
NT - MAG - Exo^{myo} III
GSEA
Volcano plot
KEGG pathway up-regulated
KEGG pathway down-regulated
GeneRatio
Count
p.adjust
Log2 fold change
−Log10 p-value
Regulation
Down-regulated
NS
Up-regulated

Supplementary Figure 7. GSEA, Volcano and KEGG pathway analysis of infiltrating macrophages isolated from mdx treated by magnetic delivery of NT-MAG-Exo^{myo}.

Dotplot visualization of Gene Set Enrichment Analysis (GSEA) and KEGG results for differentially expressed genes of Ly6C^{hi}CFSE^{hi} (iMAC). iMAC were isolated from muscles of mdx mice treated with vehicle (n=3), NT-MAG-Exo^{myo} I (n=3), NT-MAG-Exo^{myo} II (n=3) and NT-MAG-Exo^{myo} III (n=3). The GSEA dotplot visualization shows the results of Gene Set Enrichment Analysis for differentially expressed genes. The enrichment score is the Kolmogorov-Smirnov (K-S) statistic that is used to determine if the enrichment score is statistically significant. The p-values were adjusted using the Benjamini-Hochberg method, with a p-value cutoff of 0.01 and a q-value cutoff of 0.05. Each dot in the dotplot represents a specific GO category, with the dot size indicating the number of genes in the core enrichment genes of the respective gene set. The color scale indicates the significance of the enrichment analysis, with red representing higher significance and blue representing lower significance. Volcano plot depicting the differential gene expression analysis between conditions. A Genewise Likelihood Ratio Test was used for data analysis in all comparisons. The volcano plot represents the differential gene expression analysis between conditions. The x-axis represents the log2 fold change of gene expression, while the y-axis represents the negative logarithm (base 10) of the p-value The y-axis represents the raw values, as the FDR-corrected p-values may lose some information. Genes with FDR-corrected p-values < 0.05 are color-coded, with up-regulated genes in red and down-regulated genes in blue. The p-values were adjusted using the Benjamini-Hochberg method to control the false discovery rate. The KEGG dotplot analysis also utilized the one-sided version of Fisher's exact test to determine if known biological functions or processes are over-represented in the list of differentially expressed genes. In this analysis, p-values were not adjusted for multiple comparisons.

a Macrophages Veh vs NT -MAG - Exo^{myo} III

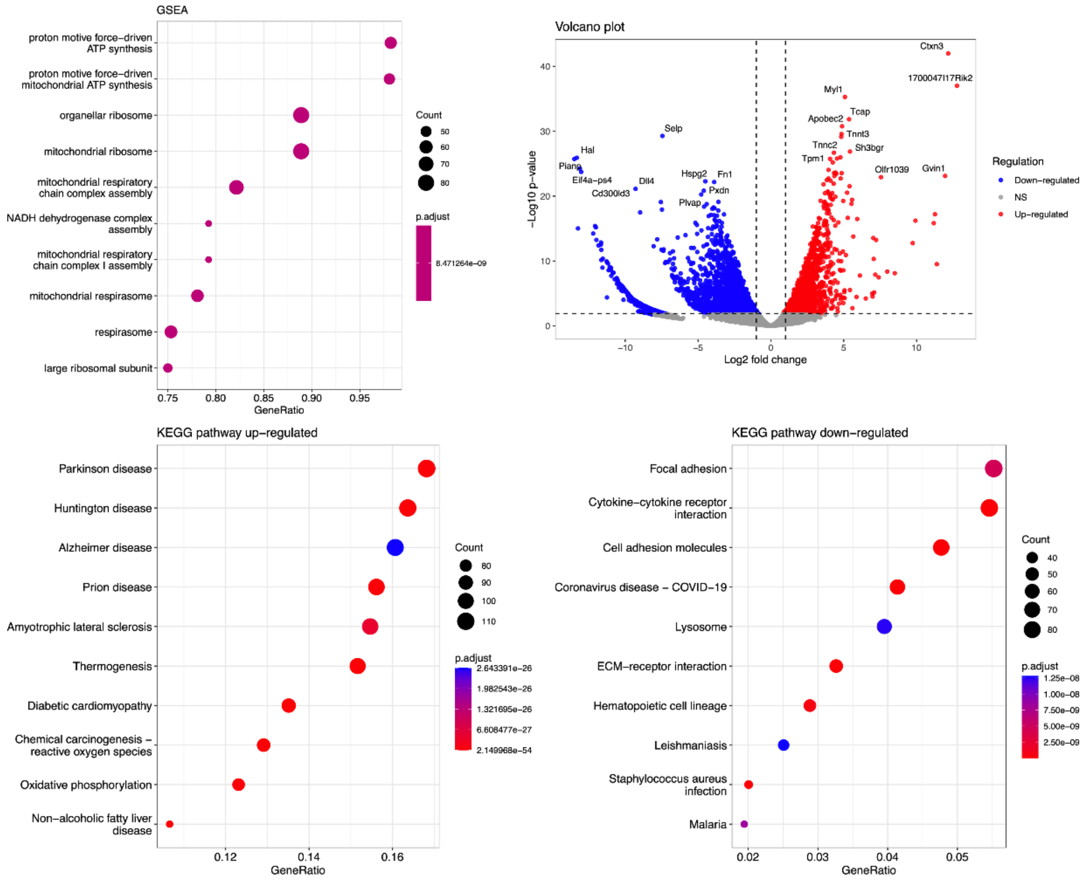

b Myofibers Veh vs NT- MAG - Exo^{myo} III

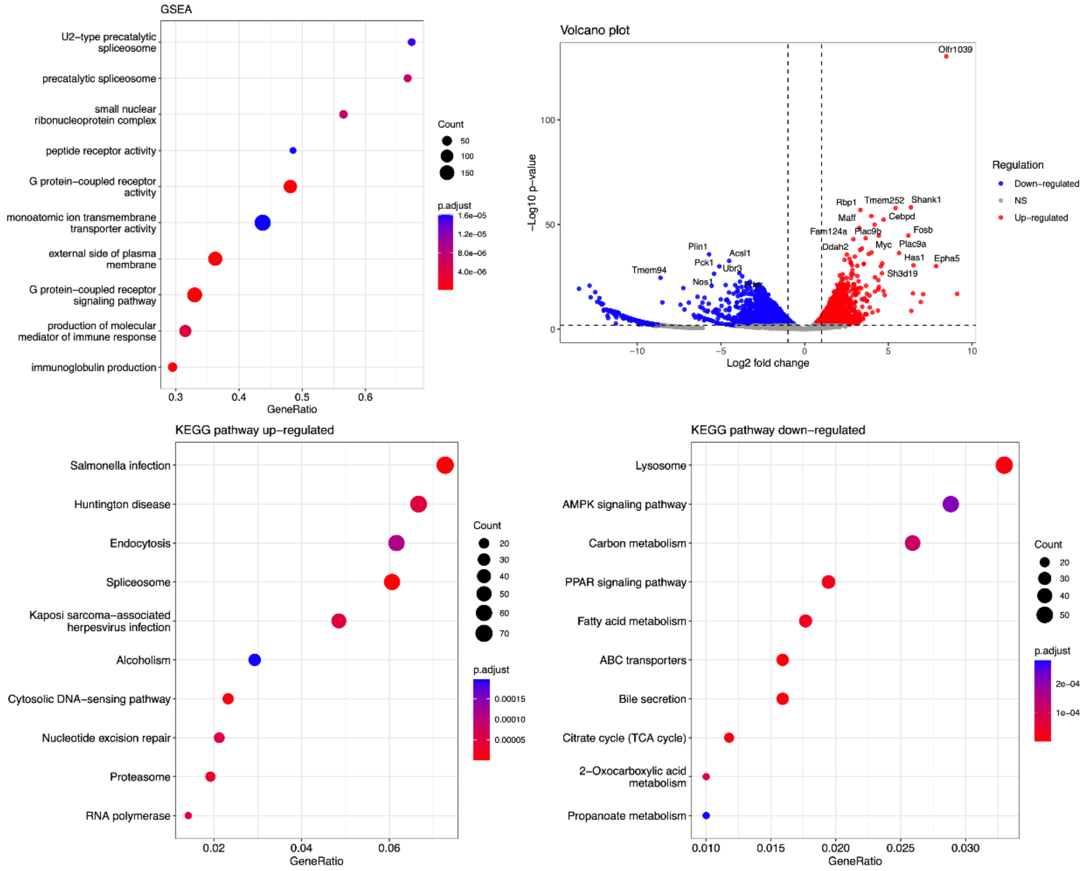

Supplementary Figure 8. GSEA, Volcano and KEGG pathway analysis of iMAC and MS isolated from mdx treated by magnetic delivery of NT-MAG-Exo^{myo}.

Dotplot visualization of Gene Set Enrichment Analysis (GSEA) and KEGG results for differentially expressed genes of iMAC (**a**) and MS (**b**) were isolated from muscles of mdx mice treated with vehicle (n=3) and NT-MAG-Exo^{myo} III (n=3). The GSEA dotplot visualization shows the results of Gene Set Enrichment Analysis for differentially expressed genes. The enrichment score is the Kolmogorov-Smirnov (K-S) statistic that is used to determine if the enrichment score is statistically significant. The p-values were adjusted using the Benjamini-Hochberg method, with a p-value cutoff of 0.01 and a q-value cutoff of 0.05. Each dot in the dotplot represents a specific GO category, with the dot size indicating the number of genes in the core enrichment genes of the respective gene set. The color scale indicates the significance of the enrichment analysis, with red representing higher significance and blue representing lower significance. Volcano plot depicting the differential gene expression analysis between conditions. A Genewise Likelihood Ratio Test was used for data analysis in all comparisons. The volcano plot represents the differential gene expression analysis between conditions. The x-axis represents the log2 fold change of gene expression, while the y-axis represents the negative logarithm (base 10) of the p-value The y-axis represents the raw values, as the FDR-corrected p-values may lose some information. Genes with FDR-corrected p-values < 0.05 are color-coded, with up-regulated genes in red and down-regulated genes in blue. The p-values were adjusted using the Benjamini-Hochberg method to control the false discovery rate. The KEGG dotplot analysis also utilized the one-sided version of Fisher's exact test to determine if known biological functions or processes are over-represented in the list of differentially expressed genes. In this analysis, p-values were not adjusted for multiple comparisons.

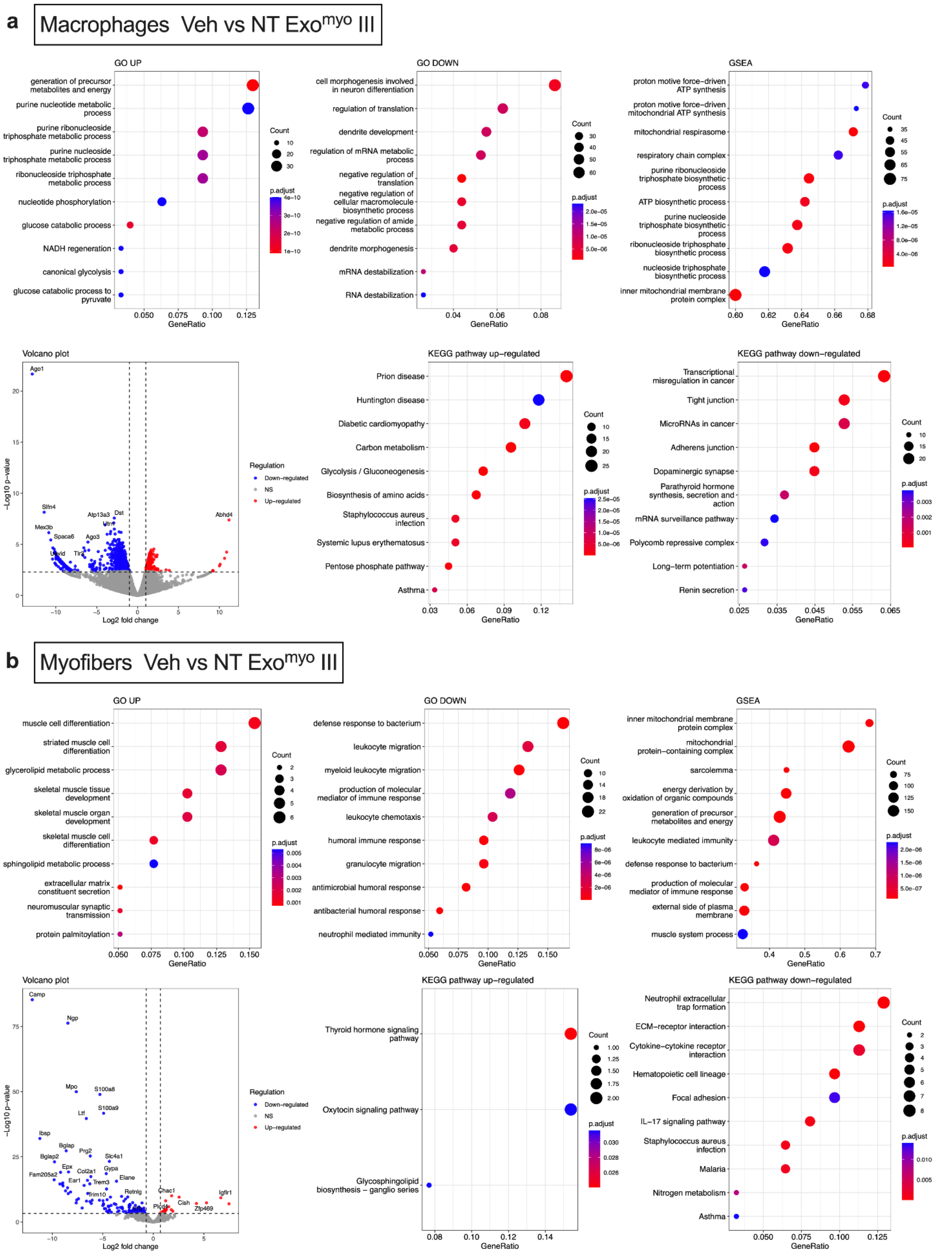
a
Macrophages Veh vs NT Exomyo III
GO UP
generation of precursor metabolites and energy
purine nucleotide metabolic process
purine ribonucleoside triphosphate metabolic process
purine nucleoside triphosphate metabolic process
ribonucleoside triphosphate metabolic process
nucleotide phosphorylation
glucose catabolic process
NADH regeneration
canonical glycolysis
glucose catabolic process to pyruvate
GeneRatio
Count
p.adjust
GO DOWN
cell morphogenesis involved in neuron differentiation
regulation of translation
dendrite development
regulation of mRNA metabolic process
negative regulation of translation
negative regulation of cellular macromolecule biosynthetic process
negative regulation of amide metabolic process
dendrite morphogenesis
mRNA destabilization
RNA destabilization
GSEA
proton motive force−driven ATP synthesis
proton motive force−driven mitochondrial ATP synthesis
mitochondrial respirasome
respiratory chain complex
purine ribonucleoside triphosphate biosynthetic process
ATP biosynthetic process
purine nucleoside triphosphate biosynthetic process
ribonucleoside triphosphate biosynthetic process
nucleoside triphosphate biosynthetic process
inner mitochondrial membrane protein complex
Volcano plot
−Log10 p−value
Log2 fold change
Regulation
Down−regulated
NS
Up−regulated
KEGG pathway up−regulated
Prion disease
Huntington disease
Diabetic cardiomyopathy
Carbon metabolism
Glycolysis / Gluconeogenesis
Biosynthesis of amino acids
Staphylococcus aureus infection
Systemic lupus erythematosus
Pentose phosphate pathway
Asthma
KEGG pathway down−regulated
Transcriptional misregulation in cancer
Tight junction
MicroRNAs in cancer
Adherens junction
Dopaminergic synapse
Parathyroid hormone synthesis, secretion and action
mRNA surveillance pathway
Polycomb repressive complex
Long−term potentiation
Renin secretion
b
Myofibers Veh vs NT Exomyo III
GO UP
muscle cell differentiation
striated muscle cell differentiation
glycerolipid metabolic process
skeletal muscle tissue development
skeletal muscle organ development
skeletal muscle cell differentiation
sphingolipid metabolic process
extracellular matrix constituent secretion
neuromuscular synaptic transmission
protein palmitoylation
GO DOWN
defense response to bacterium
leukocyte migration
myeloid leukocyte migration
production of molecular mediator of immune response
leukocyte chemotaxis
humoral immune response
granulocyte migration
antimicrobial humoral response
antibacterial humoral response
neutrophil mediated immunity
GSEA
inner mitochondrial membrane protein complex
mitochondrial protein−containing complex
sarcolemma
energy derivation by oxidation of organic compounds
generation of precursor metabolites and energy
leukocyte mediated immunity
defense response to bacterium
production of molecular mediator of immune response
external side of plasma membrane
muscle system process
Volcano plot
KEGG pathway up−regulated
Thyroid hormone signaling pathway
Oxytocin signaling pathway
Glycosphingolipid biosynthesis – ganglio series
KEGG pathway down−regulated
Neutrophil extracellular trap formation
ECM−receptor interaction
Cytokine−cytokine receptor interaction
Hematopoietic cell lineage
Focal adhesion
IL−17 signaling pathway
Staphylococcus aureus infection
Malaria
Nitrogen metabolism
Asthma

Supplementary Figure 9. GSEA, Volcano and KEGG pathway analysis of iMAC and MS isolated from mdx treated by magnetic delivery of NT-Exo^{myo}.

Dotplot visualization of Gene Set Enrichment Analysis (GSEA) and KEGG results for differentially expressed genes of iMAC (**a**) and MS (**b**) were isolated from muscles of mdx mice treated with vehicle (n=3) and NT-Exo^{myo} III (n=3). The GSEA dotplot visualization shows the results of Gene Set Enrichment Analysis for differentially expressed genes. The enrichment score is the Kolmogorov-Smirnov (K-S) statistic that is used to determine if the enrichment score is statistically significant. The p-values were adjusted using the Benjamini-Hochberg method, with a p-value cutoff of 0.01 and a q-value cutoff of 0.05. Each dot in the dotplot represents a specific GO category, with the dot size indicating the number of genes in the core enrichment genes of the respective gene set. The color scale indicates the significance of the enrichment analysis, with red representing higher significance and blue representing lower significance. Volcano plot depicting the differential gene expression analysis between conditions. A Genewise Likelihood Ratio Test was used for data analysis in all comparisons. The volcano plot represents the differential gene expression analysis between conditions. The x-axis represents the log2 fold change of gene expression, while the y-axis represents the negative logarithm (base 10) of the p-value The y-axis represents the raw values, as the FDR-corrected p-values may lose some information. Genes with FDR-corrected p-values < 0.05 are color-coded, with up-regulated genes in red and down-regulated genes in blue. The p-values were adjusted using the Benjamini-Hochberg method to control the false discovery rate. The KEGG dotplot analysis also utilized the one-sided version of Fisher's exact test to determine if known biological functions or processes are over-represented in the list of differentially expressed genes. In this analysis, p-values were not adjusted for multiple comparisons.

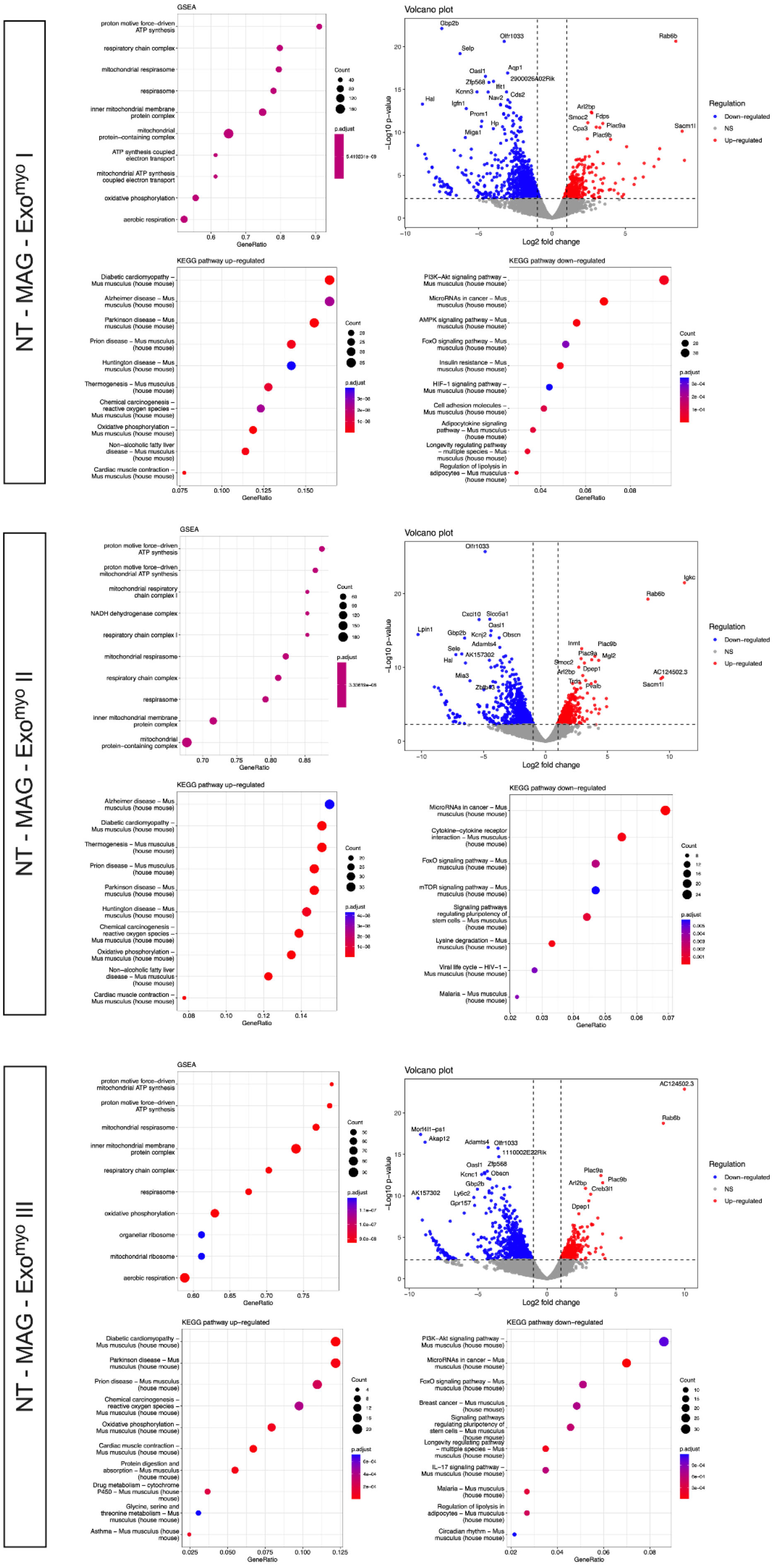

NT - MAG - Exomyo I
GSEA
Count
p.adjust
GeneRatio
Volcano plot
Log2 fold change
−Log10 p−value
Regulation
Down−regulated
NS
Up−regulated
KEGG pathway up−regulated
KEGG pathway down−regulated
NT - MAG - Exomyo II
GSEA
Volcano plot
KEGG pathway up−regulated
KEGG pathway down−regulated
NT - MAG - Exomyo III
GSEA
Volcano plot
KEGG pathway up−regulated
KEGG pathway down−regulated

Supplementary Figure 10. GSEA, Volcano and KEGG pathway analysis of infiltrating macrophages isolated from mdx treated by magnetic delivery of NT-MAG-Exo^{myo}.

Dotplot visualization of Gene Set Enrichment Analysis (GSEA) and KEGG results for differentially expressed genes of MS isolated from muscles of mdx mice treated with vehicle (n=3), NT-MAG-Exo^{myo} I (n=3), NT-MAG-Exo^{myo} II (n=3) and NT-MAG-Exo^{myo} III (n=3). The GSEA dotplot visualization shows the results of Gene Set Enrichment Analysis for differentially expressed genes. The enrichment score is the Kolmogorov-Smirnov (K-S) statistic that is used to determine if the enrichment score is statistically significant. The p-values were adjusted using the Benjamini-Hochberg method, with a p-value cutoff of 0.01 and a q-value cutoff of 0.05. Each dot in the dotplot represents a specific GO category, with the dot size indicating the number of genes in the core enrichment genes of the respective gene set. The color scale indicates the significance of the enrichment analysis, with red representing higher significance and blue representing lower significance. Volcano plot depicting the differential gene expression analysis between conditions. A Genewise Likelihood Ratio Test was used for data analysis in all comparisons. The volcano plot represents the differential gene expression analysis between conditions. The x-axis represents the log2 fold change of gene expression, while the y-axis represents the negative logarithm (base 10) of the p-value The y-axis represents the raw values, as the FDR-corrected p-values may lose some information. Genes with FDR-corrected p-values < 0.05 are color-coded, with up-regulated genes in red and down-regulated genes in blue. The p-values were adjusted using the Benjamini-Hochberg method to control the false discovery rate. The KEGG dotplot analysis also utilized the one-sided version of Fisher's exact test to determine if known biological functions or processes are over-represented in the list of differentially expressed genes. In this analysis, p-values were not adjusted for multiple comparisons.

Antibodies	Identifier	Dilution	Source
Rabbit Anti mouse sarcomeric alpha actin	ab68167	1:500	Abcam
Rat Anti CD68 Alexa-Fluor 647	AM ab201845	1:100	Abcam
Rabbit Anti mouse laminin	L9393	1:50	Sigma-Aldrich
Rabbit Anti-mouse CD206	E6T5J	1:100	Cell Signaling
Mouse Anti-embryonic Myosin Heavy Chain	F 1.652	1:50	Developmental Studies Hybridoma Bank
Goat Anti-IgG mouse Alexa Fluor PLUS 488-conjugated antibody	A-21121	1:100	Invitrogen
Rabbit Anti-mouse desmin	Ab15200	1:200	Abcam
DAPI	28718-90-3	1:1000	Sigma-Aldrich
CD9 Recombinant Rabbit Monoclonal Antibody (SA35-08)	MA5-31980	1:600	Thermo Fisher Scientific
Antibody anti-CD81 Mouse Monoclonal IgG1	SC-7637	1:100	Santa Cruz Technologies
Antibody anti-CD63 Mouse Monoclonal IgG1	SC-5275	1:200	Santa Cruz Technologies
Antibody anti-TSG 101 Mouse Monoclonal IgG1 clone 4a10	ab83	1:600	Abcam
Antibody anti -ALIX (3A9) Mouse Monoclonal IgG1	SC-53538	1:600	Santa Cruz Technologies
Antibody Rabbit Policlonal Anti-trimethyl Histone H3 (Lys27)	ABE44	1:600	Merck Millipore
Antibody Mouse Monoclonal [16D10AD9AH5] to Ubiquinol-Cytochrome C Reductase Core Protein I	ab110252	1:600	Abcam
Antibody Rabbit Polyclonal IgG anti-AGO-2	10686-1-ap	1:600	Proteintech
Antibody anti-Annexin A1 Rabbit Polyclonal IgG	71-3400	1:600	Invitrogen, Thermo Fisher Scientific
MYOD Mouse Monoclonal IgG1 Antibody (5.8A)	MA512902	1:600	Invitrogen, Thermo Fisher Scientific
TGFb1 Rabbit Polyclonal Antibody	ab33090	1:600	Elabscience
Recombinant Mouse TNF-alpha/TNFA Protein	PKSM041176	1:600	Elabscience
Recombinant Anti-NF-kB p65 antibody [E379] Rabbit Monoclonal	ab32536	1:600	Abcam
Antibody Relb (D-4) Mouse Monoclonal IgG1	sc-48366	1:600	Santa Cruz Technologies
Antibody MMP9 (E-11) Mouse Monoclonal IgG	sc-393859	1:600	Santa Cruz Technologies
Antibody IL-6 (10E5) Mouse Monoclonal IgG2bk	sc-57315	1:600	Santa Cruz Technologies
Antibody NFkB/IkBa (H-4) Mouse Monoclonal IgG1k	sc-1643	1:600	Santa Cruz Technologies
Antibody TLR4 (25) Mouse Monoclonal IgG1k	sc-293072	1:600	Santa Cruz Technologies
Recombinant anti-AKT1-2-3 (ERP16798) Rabbit Monoclonal	ab179463	1:600	Abcam
Antibody anti-mTOR [EPR390(N)] Rabbit Monoclonal	AM ab134903	1:600	Abcam
Antibody HMGB1 HAP46.5 Mouse Monoclonal IgG1k	sc-56698	1:600	Santa Cruz Technologies
S6 Ribosomal Protein (S6RP) (5G10) Rabbit IgG	2217S	1:600	cell signaling
Total OXPHOS antibody Cocktail	MS604-300	1:600	Abcam
Antibody anti-CD206 Rabbit Polyclonal IgG	ab64693	1:600	Abcam
Antibody anti-Pax7 Rabbit Polyclonal IgG [EPR3353]	ab92317	1:600	Abcam
Antibody anti-ATG7 Mouse Monoclonal	sab4200304	1:600	Sigma-Aldrich
Antibody anti-p62 SQSTM1 (C-terminal) Rabbit Polyclonal	p0068	1:600	Sigma-Aldrich
Antibody anti-LC3B	L7543	1:600	Sigma-Aldrich
Antibody antti-4E-BP1 Goat Polyclonal IgG	AF3227	1:600	R&D
Antibody anti-MyD88 Rabbit Polyclonal	23230-I-AP	1:600	Proteintech
Polyclonal Rabbit anti-Goat IgG HRP	P0160	1:1000	DAKO
Polyclonal Swine anti-Rabbit Immunoglobulins HRP	P0217	1:1000	DAKO
Polyclonal Rabbit anti-Mouse IgG HRP	P0260	1:1000	DAKO
CD45 Antibody, FITC, Anti Mouse	130-110-658	Working dilution: 0.2 mg/mL	Miltenyi Biotec
CD45 Antibody, BV510, Anti Mouse	103137	Working dilution: 0.2 mg/mL	BioLegend
CD31 Antibody FITC, Anti Mouse	130-123-675	Working dilution: 0.2 mg/mL	Miltenyi Biotec
F4/80 Antibody, FITC, Anti-Mouse	130-117-509	Working dilution: 0.2 mg/mL	Miltenyi Biotec
F4/80 Antibody, APC, Anti-Mouse	123115	Working dilution: 0.2 mg/mL	BioLegend
Anti- alpha7 integrin (ITGA7) Antibody , PE-Vio770, Anti-Mouse	130-103-357	Working dilution: 0.5 mg/mL	Miltenyi Biotec
Sca1 Antibody, PE, Anti Mouse	130-118-073	Working dilution: 0.5 mg/mL	Miltenyi Biotec
CD34 Antibody, APC, Anti-Mouse	MA546841	Working dilution: 0.5 mg/mL	Thermo Fisher Scientific
7AAD	559925	Working dilution: 0.5 mg/mL	BD Biosciences
CD11b Antibody, PE, Anti-Mouse	101207	Working dilution: 0.5 mg/mL	BioLegend
CD68 Antibody, APC, Anti-Mouse	130-112-857	Working dilution: 0.5 mg/mL	Miltenyi Biotec
CD9 Antibody, APC Cy7, Anti-Mouse	ab239306	Working dilution: 0.5 mg/mL	Abcam
CD81 Antibody, PE Cy7, Anti-Mouse	NBP1-44861PECY7	Working dilution: 0.5 mg/mL	Novus Biologicals
IgG1k Antibody, PE, Anti-Mouse	400111	Working dilution: 0.2 mg/mL	Biolegend
Ly6C Antibody, PE Cy7, Anti-Mouse	128017	Working dilution: 0.5 mg/mL	Biolegend
ExoFlow 2.0 CD63 kit for tissue culture media	EXOFLOW300A-1	n/a	SBI, System Biosciences

Supplementary Table 1

Chemicals	Identifier	Source
DMEM high glucose	11960-044	Gibco, Thermo Fisher Scientific
Xylene	1330-20-7	Sigma-Aldrich, Merck
Alexa Fluor™ 647 Carboxylic Acid, tris(triethylammonium) salts	A33O84	Invitrogen, Thermos fisher scientific
Amphotericina B	ECM 0009D	Euroclone
CFSE	C34554	Invitrogen
Clarity Western ECL Substrate	170-5061	Biorad
Collagenase II	17101-015	Gibco, Thermo Fisher Scientific
dichloromethane	75-09-2	Sigma-Aldrich, Merck
Diphenyl ether (DPE)	101-84-8	Sigma-Aldrich, Merck
Dispase	17105-041	Gibco
DPX reagent	MERC1.00579.0500_P	VWR International, Avantor, Merck
F10	11550-043	Gibco, Thermo Fisher Scientific
Donkey serum	566460	Sigma-Aldrich, Merck
Goat Serum	G9023	Sigma-Aldrich, Merck
Hydroxyproline Assay Kit	MAK008	Sigma-Aldrich, Merck
Iron(III) acetylacetonate1-dodecanediol (DCD)	14024-18-1	Sigma-Aldrich, Merck
Laemmli sample buffer 4X	1610747	Biorad
LDS Sample Buffer non-reducing 4x	84788	Thermo Fisher Scientific
Liberase	5401020001	Roche
$MgCl_2$	7786-30-3	Sigma-Aldrich, Merck
microBCA Protein Assay Kit	23235	Thermo Fisher Scientific
Na2SiO3 * 9H2O	13517-24-3	Sigma-Aldrich, Merck
NaOH	1310-73-2	Sigma-Aldrich, Merck
N-hexane	110-54-3	Sigma-Aldrich, Merck
Nitro-blue tetrazolium	298-83-9	Sigma-Aldrich, Merck
Oleic acid	112-80-1	Sigma-Aldrich, Merck
Oleylamine	112-90-3	Sigma-Aldrich, Merck
Optiprep	7820	Stem Cell Technologies
Penicillin/Streptomycin	DE17-602E	Lonza Bioscence
PFA	28908	Thermo Fisher Scientific
Phosphate buffer	7778-77-0	Thermo Fisher Scientific
RNeasy Micro kit	74004	Quiagen
RPMI 1640	ECM0495L	Euroclone
Tetramethyl ammonium 11-aminoundecanoate TAU	947601-83-4	Sigma-Aldrich, Merck
Triton-X 100	T9284	Sigma-Aldrich, Merck
FastStart Universal SYBR Green Master	4913949001	Roche
TrueVIEW Autofluorescence Quenching Kit	SP-8400	Vector Laboratories
**Set up**		
Optima XE, Ti 45 fixed-angle rotor	A94471	Beckman Coulter
NanoSight NS300		Malvern Panalytical, Spectris
Branson Sonifier	101-063-966R	Branson, SFX250
cHiPLC-nanoflex system		Eksigent Technologies, AB SCIEX
Q-Exactive mass spectrometer		Thermo Fisher Scientific
Leica DMIRE2		Leica Biosystems
MiniMACS Separator		Miltenyi Biotec
LS column		Miltenyi Biotec
BD Canto II machine		BD Biosciences
T Bruker Minispec mq20 broadband spectrometer		Bruker
Glomax microplate reader	GM3000	Promega
Bruker 2D U-OI		Ultra-Optical Imaging
7T MRI scanner		Bruker, BioSpec 70/30 USR, Paravision 5.0
Transparent treadmill belt		Clever Sys
Leica Laser microdissector (CTR6000).		Leyca Microsystem
AGILENT Tapestation 2200 (high sensitivity RNA chip)		AGILENT
7900HT Fast Real-Time PCR System	4329001	Applied Biosystems Thermo Fisher Scientific

Supplementary Table 2

Primers	Forward	Reverse
iNOS	5’-GTTCTCAGCCCAACAATACAAGA-3’	5’-GTGGACGGGTCGATGTCAC-3’
CxC110	5’-CCAAGTGCTGCCGTCATTTTC-3’	5’-GGCTCGCAGGGATGATTTCAA-3’
CD86	5’-GGCTCGCAGGGATGATTTCAA-3’	5’-TTGAGCCTTTGTAAATGGGCA-3’
Tnfα	5’-CCCTCACACTCAGATCATCTTCT-3’	5’-GCTACGACGTGGGCTACAG-3’
Ym1	5’-CAGGTCTGGCAATTCTTCTGAA-3’	5’-GTCTTGCTCATGTGTGTAAGTGA-3’
CCL22	5’-AGGTCCCTATGGTGCCAATGT-3’	5’-CGGCAGGATTTTGAGGTCCA-3’

Supplementary Table 3

References

1 Florio, F., Accordini, S., Libergoli, M. & Biressi, S. Targeting Muscle-Resident Single Cells Through in vivo Electro-Enhanced Plasmid Transfer in Healthy and Compromised Skeletal Muscle. *Frontiers in physiology* 13, 834705, doi:10.3389/fphys.2022.834705 (2022).

2 Parolini, D. *et al.* CD20-related signaling pathway is differently activated in normal and dystrophic circulating CD133(+) stem cells. *Cellular and molecular life sciences : CMLS* 66, 697-710, doi:10.1007/s00018-009-8652-2 (2009).

3 Jung, J. M. *et al.* Reference intervals for whole blood viscosity using the analytical performance-evaluated scanning capillary tube viscometer. *Clinical biochemistry* 47, 489-493, doi:10.1016/j.clinbiochem.2014.01.021 (2014).

4 Robbins, N., Thompson, A., Mann, A. & Blomkalns, A. L. Isolation and excision of murine aorta; a versatile technique in the study of cardiovascular disease. *Journal of visualized experiments : JoVE*, e52172, doi:10.3791/52172 (2014).
